# Advanced Research and Development of Face Masks and Respirators Pre and Post the Coronavirus Disease 2019 (COVID-19) Pandemic: A Critical Review

**DOI:** 10.3390/polym13121998

**Published:** 2021-06-18

**Authors:** Ebuka A. Ogbuoji, Amr M. Zaky, Isabel C. Escobar

**Affiliations:** 1 Department of Chemical and Materials Engineering, University of Kentucky, Lexington, KY 40506, USA; ebuka.ogbuoji@uky.edu; 2 BioMicrobics Inc., 16002 West 110th Street, Lenexa, KS 66219, USA; azaky@biomicrobics.com

**Keywords:** coronavirus, *SARS-CoV-2*, personal protection equipment, aerosol, filtration

## Abstract

The outbreak of the COVID-19 pandemic, in 2020, has accelerated the need for personal protective equipment (PPE) masks as one of the methods to reduce and/or eliminate transmission of the coronavirus across communities. Despite the availability of different coronavirus vaccines, it is still recommended by the Center of Disease Control and Prevention (CDC), World Health Organization (WHO), and local authorities to apply public safety measures including maintaining social distancing and wearing face masks. This includes individuals who have been fully vaccinated. Remarkable increase in scientific studies, along with manufacturing-related research and development investigations, have been performed in an attempt to provide better PPE solutions during the pandemic. Recent literature has estimated the filtration efficiency (FE) of face masks and respirators shedding the light on specific targeted parameters that investigators can measure, detect, evaluate, and provide reliable data with consistent results. This review showed the variability in testing protocols and FE evaluation methods of different face mask materials and/or brands. In addition to the safety requirements needed to perform aerosol viral filtration tests, one of the main challenges researchers currently face is the inability to simulate or mimic true aerosol filtration scenarios via laboratory experiments, field tests, and in vitro/in vivo investigations. Moreover, the FE through the mask can be influenced by different filtration mechanisms, environmental parameters, filtration material properties, number of layers used, packing density, fiber charge density, fiber diameter, aerosol type and particle size, aerosol face velocity and concentration loadings, and infectious concentrations generated due to different human activities. These parameters are not fully understood and constrain the design, production, efficacy, and efficiency of face masks.

## 1. Introduction

The unprecedented outbreak of the coronavirus disease 2019 (COVID-19) has caused the spread of Severe Acute Respiratory Syndrome Coronavirus (*SARS-CoV-2*) that is currently a global concern. *SARS-CoV-2* has a mortality rate of 3 to 5% and can cause severe pneumonia, acute myocardial injuries, and chronic damage to the cardiovascular system [1]. The lack of knowledge and incomplete understanding of COVID-19 limits current advancements in research, product development, and manufacturing of respirators and face masks. Prior research into fabric masks dates back in history to the 1918–1920 *H1N1 Influenza A virus* pandemic, known as the Spanish Flu. However, since the COVID-19 outbreak, there has been a surge in conducting applied research and development to improve face masks and respirators, facilitate standard testing approvals, accept new standard and nonstandard practices, and accelerate the certification process for newly developed products. For instance, the use of expired respirators and the application of various decontamination processes have been accepted for use since March 2020, in order to prolong the use of respirators and face masks [2]. Face mask is a term to express a wide range of face protective equipment that can reduce the transmission of infectious droplets. Surgical masks are intent to protect patients with open wounds against possible surrounding infectious agents during surgical procedures. Currently, due to the demand on face masks, surgical masks have been examined for their applicability in preventing the transmission of the human coronavirus and the *influenza* virus from symptomatic individuals [3].

Our existing knowledge of respiratory infections such as *influenza*, *SARS-CoV-1*, and *MERS-CoV* cannot provide a full and clear understanding of the current novel coronavirus. Similar to *influenza* viruses, new strains emerge and can cause a global pandemic [4,5]. *SARS-CoV-2* virions have been reported to range from 60 to 140 nm in size with an average size of 125 nm. The virions are carried via respiratory droplets with sizes ranging from 0.1 to 1000 µm [6]. The novel coronavirus can be transmitted by small and large droplets taking into consideration that small droplets provide higher risk than large droplets as they can remain airborne for extended durations. Literature is not completely consistent on describing the size distribution of particles generated from breathing, coughing, and sneezing [7]. In addition, previous knowledge gained from theoretical and experimental mechanistic studies on aerosol filtration by fibrous media is not immediately applicable in determining the blocking mechanisms of viral particles. It is thought that the routes of transmission are due to the spread of aerosols and respiratory droplets containing virus particles [8]; however, there have been cases where transmission has occurred to patients in which the route of transmission could not be tracked. Furthermore, it has been reported that infections with the coronavirus have been reported in individuals that did not have relevant travel history or exposure to another person with COVID-19, suggesting that the route of exposure was through community transmission. Studies have shown evidence of transmission from asymptomatic and pre-symptomatic COVID-19 patients [9,10].

After the outbreak of COVID-19, the market has been saturated with uncertified commercially available and home-made face masks. These masks are fabricated with materials that have not been tested or have been tested under specific testing parameters that may not be representative of the accurate means of protection, transmission mechanism(s), fitting and leakage-mitigating conditions, FE under non-ideal conditions, and considerations for social and environmental factors. Furthermore, there have been claims that these masks have not been tested in practice [11]. During the 1918–1920 pandemic, multiple layers of cloth masks were tested using a series of experiments using controlled sprays and real coughing to create bioaerosols [7]. Recently, there have been numerous studies trying to evaluate the FE of home-made face masks made from different types of fabric materials including cloth masks. It must be understood that, during this unprecedented outbreak, innovative solutions, practices and case studies performed to produce more efficient and effective filters are appreciated, but each case study has its own limitations and constraints on its evaluation. For instance, most of the existing data on the FE of face masks and respirators were collected from vitro experiments with non-biological particles, which may not be representative of infectious respiratory virus droplets [3]. Moreover, these limitations are expected during the pandemic due to the lack of knowledge in understanding the novel coronavirus characteristics, its viability, routes, and rates of transmissions. In addition, there is lack of knowledge in understanding the proper filtration material(s) per application, governing filtration parameters, accurate testing protocols, usage of masks under non-ideal conditions and for extended periods, conditions for fitting tests and root causes of leakage, regeneration of masks and their decontamination processes, and other environmental variables. Although home-made masks do not provide the same level of protection as surgical masks and respirators, the CDC has recommended using fabric face masks as a short-term alternative solution. The primary goal of this recommendation is to limit the spread of viral particles due to respiratory activities rather than providing efficient blockage of contagious particles to the face mask wearer [12,13]. More specifically, it is suggested that multilayered masks may increase the level of protection against nanometer-sized aerosols. Therefore, from a public policy perspective, the majority of states in the United States and more than 130 nations have issued guidelines either requiring or recommending wearing masks, regardless of their material, in public settings to mitigate the spread of COVID-19 [14]. The (WHO) estimated in March 2020 that 89 million masks would be needed each month. In addition, due to the lack of supply and affordability of face masks, the WHO has also changed its position from “not recommended under any circumstance” to “there is no current evidence to make a recommendation for or against their use” [7].

There is also a critical knowledge gap in understanding the dependency of filter material properties and mask fit on the evaluation of masks’ FE. Several research groups have tested different filter materials and measured the FE in ideal-fit scenarios without consideration for leakage [13]. Fit tests measure total aerosol penetration occurring through the filter medium and through the face-seal leaks. However, under actual breathing conditions, none of the currently performed standard methods or tests account clearly between penetration through face-seal leakage and penetration through filter medium [15]. It is also not really understood the difference between measurements taken using qualitative and quantitative testing methods. Therefore, determining mask efficacy is a complex topic and an active field of research.

It is expected that while daily practices and lifestyle can be altered during and after the pandemic, there is a need in finding alternative solutions in order to function without spreading COVID-19, especially during any human-to-human interaction. Providing the essential level of protection is currently crucial for medical staff and first responders; however, the *SARS-CoV-2* outbreak has left many communities without sufficient quantities of face masks. Moreover, in addition to maintaining social distancing and constantly washing hands, wearing face masks is becoming a global necessity for individuals who may live and/or work in public settings such as hospitals, public offices, buildings, trains, supermarkets, and shopping malls. Unfortunately, the supply of commercially certified respirators and face masks has not met the demand and/or has not provided more affordable options especially in low-resource areas and for people living in poverty. Furthermore, even when surgical face masks and respirators are available, there are concerns about their side effects and discomfort of prolonged use [11].

## 2. Historical Development of Aerosols Face Protection

### 2.1. Face Masks

The use of face mask can be traced back to the 13th century when silk scarves were used, by Chinese servants, for covering their mouths and noses to avoid contaminating the emperor’s meal [16]. The plague, which tormented Europe in the 17th century, led to the use of facial mask as a protective gear against microbes. During this time, the French doctor Charles de Lorme designed a beak-like mask for protection from the outbreak [17]. The beak-like mask worn by plague physicians had provisions for a theriac, which was composed of more than fifty-five herbs, to combat the infectious miasma thought to cause the disease. The beak-like design ensured sufficient time for the protective herbs to purify the contaminated air before it reached the nose and lungs [17,18].

The dawn of the 20th century saw the first recorded use of a face covering for medical purposes by the German physician Johannes Von Mikulicz in 1897. This was reported in a collaborative work between Mikulicz and the German clinician Carl Friedrich Flügge. While investigating tuberculosis, Flügge conceived his droplet theory of infection, which suggested using facemasks to prevent the spread of microbes [19,20]. The face masks recorded in their publication, which was a single-layered mask made of gauze, covered only the mouth and were described as a “mouth bandage” [20,21]. In 1898, Hübner who was Mikulicz’s assistant, performed and published a study showing the effect of face masks on droplet spread. He described a two-layer mouth protection made of gauze, which prevents the spread of droplets [20,22]. Seven years later, it was shown that wearing a “mouthguard” held back sputum droplets that played a vital role in the spread of tuberculosis [23,24]. Face masks were used only to cover the wearer’s mouth for infection prevention until 1918 when Dr. George H. Weaver and his group started covering both mouth and nose to protect the wearer [25,26]. In 1937, earing of masks over the mouth alone was later proven to be inadequate providing full protection [27]. Weaver also recommended that face masks be worn twice except after sterilization [26], and their results also inspired more experimental studies to determine the most effective type of face masks. The first reported mask efficiency experiments were performed in 1918, when coarse gauze, medium gauze, and butter cloth masks were challenged against a gargled solution of *Bacillus prodigiosus*, and it was observed that the finer the gauze, the more efficient the mask [26]. Others also conducted efficiency experiments and concluded that mask efficiency was a direct ratio of weave density and gauze thickness, and that the distance traveled by droplets in air depended on the force with which the droplets were released [23].

Despite improvements in face masks, multilayer cotton masks were found to be inefficient in preventing the spread of the 1918 Spanish flu because of poor mask quality and inappropriate use [25]. This led to a decade long development of more efficient and comfortable masks. Some notable masks made during this time included the Dannenburg mask which was made up of a galvanized wire mesh cut to fit the face and adjusted to fit the nose with a double layered gauze placed on the wire mesh and held tight using paper clips [26]. The Mellinger mask was built using a 14-carat gold-filled wire mask support adjusted to fit the nose and hung on the ear with newspaper or wax paper hung on the support and extended to the chin [28]. The Walker mask was made of regular gauze mesh support, which had a thin piece of rubber placed between the gauze layers. The Blatt and Dale mask was a standard gauze mask that had cellophane placed between the gauze layers. The Walter mask was like Walker mask but had a cellulosic derivative, plastacele, placed in between the gauze, and it was held in place by cotton ties with an aluminum band at the nose [26]. These masks operated mainly on the principle of deflection; however, the filter type mask was preferred because it reduced the amount of bacteria in the room [23].

The invention of new polymeric materials in early 1950s paved the way for more efficient and cost-effective mask materials. The first disposable mask made up of a glass fiber mat material with a thickness of 0.06 inch to 0.08 inch that could remove up to 97% of microbes from an aerosol was invented in 1967 [23]. New testing methods were then developed to determine the efficiency of face masks by replacing humans with mannequins [23]. The tests conducted on new mask materials showed that polypropylene and polyester rayon fibers’ efficiencies outperformed cellulose and glass fiber mats [23]. This introduced the new era of mask materials, which has led to various materials being used today.

### 2.2. Respirators

Respirators were initially designed to combat the inherent hazards of mining. The first recorded use of a respirator was in the first century AD when Gaius Plinius Secundus, a Roman naturalist, suggested using animal bladder to protect Roman miners from inhaling lead oxide dust [29]. The prominent Leonardo da Vinci, in the 16th century, proposed using a wet cloth as a facial covering to protect against toxic chemicals [30,31]. Later, the industrial revolution in the early 1800s caused other environmental concerns that made more sophisticated respirators necessary [31]. The ability to distinguish between the nature of dust and gaseous contaminants, a recent discovery during that time, was vital in designing and improving respirators to tackle rising environmental concerns. Furthermore, early respirators were also designed to help firefighters [31]. In 1825, John Roberts developed a respirator for firefighters with a leather hood and a hose strapped to the leg. The hose had an inverted funnel containing coarse woolen cloth and a moist sponge for water-soluble gases and vapor removal. Activated charcoal was later introduced in firefighters’ respirators after it was observed to have the ability to remove organic vapors and gases from the air [31]. The discovery of Brownian Motion by Robert Brown in 1827 motivated the use of masks for protection against dust particles, which further led to the improvement of respirator design [16]. Lewis Haslett, an American inventor, filed the first patent for a lung respirator for miners in 1849, which was composed of two one-way clapper valves and moisten wool as a filter material [30].

The chemical warfare in World War I (WWI) caused a drastic increase in respirator research and development. A respirator with pads engulfed with activated charcoal and immersed in bicarbonate and sodium thiosulfate was developed in Germany. While in Britain, a respirator with an expiratory valve and a “small box respirator” with a tube mouthpiece connected to a canister containing neutralizing chemicals was developed [32]. Towards the end of WWI, the US military took a keen interest in respirators for defense against chemical warfare with the development of the American Small Box Respirator (ASBR). It was produced from rubber with elliptical eye holes made from tri-flex safety material to address the issue of lens fogging by channeling the incoming air over the eyepieces [33]. Improved versions of the ASBR, namely, MIA1 and MIA2 service gas masks, were developed with detachable lenses and having the inlet valve positioned differently than the original ASBR [33].

Respirators were first used in the US health care sector in late 1980s when the number of *Mycobacterium tuberculosis* (TB) cases increased despite the use of surgical masks. Occupational Safety and Health Association (OSHA) and the CDC instituted guidelines and recommendations on respiratory protection in 1997, which led to the development of single-use, effective, and affordable respirators for TB protection like the N95 respirator [34,35].

## 3. Patents in Face Masks and Respirators

In this section, published patents to enhance the efficacy and efficiency of face masks and respirators for aerosol particle filtration are discussed. It must be noted that only US patents approved by the United States Patent and Trademark Office and European patents approved by the European Patent Office are included here.

Early face mask patents awarded to inventors, in the United States, had a major limitation on allowing the passage of aerosols through the gap between the mask and the wearer’s face [36,37]. Aerosols can contain pathogens which could infect the wearer; hence, affecting the mask efficacy. In a patent published in December 1997, George et al. [38] attempted to tackle this challenge by designing a shield or visor that could be mounted on a surgical mask to prevent the passage of fluids between the mask periphery and the wearers face. Vance et al. [39] also patented a design with a shield visor attached to a mask to prevent the passage of liquids from the mask exterior to the wearer’s face.

Some effective masks were invented by modifying the mask shape, sealing method, and filter material [38,40,41,42]. These modified mask designs showed high efficiency in protecting the wearer but have suffered from fogging of the wearers’ eyeglasses. Lauer et al. [43] attempted to solve this problem by using an air-impervious material placed at the top of the mask inner or outer surface, which could also be placed on both the inner and outer surfaces. In another attempt, Cox et al. [44] described a face mask with an opening covered with a perforated filter material to facilitate movement of exhaled moist air leading to enhanced breathability and glass fogging prevention. Facer et al. [45] described an altered intrinsic structure of the sinus region of a face mask to increase resistance and reduce fogging. Lastly, Bora et al. used properly positioned vents on the face mask to allow for the removal of exhaled air laterally instead of going upwards [46].

Other modifications have been accomplished to increase the efficiency and comfort of face masks. Japuntich et al. [47] described a face mask with an exhalation valve containing at least one orifice, which allowed exhaled air flow from interior gas space to an exterior gas space. In addition, an exhale filter element was included in the mask to capture contaminants. Moreover, masks with proposed better fit to eliminate contamination associated with loose face masks have been invented [48]. Gloag et al. [49] presented a design of respirators that could be opened and used without touching the interior surface; thereby, preventing contamination. Welchel et al. [50] invented a respirator with worn straps and exhalation vents to prevent fogging. The straps used were proposed to have “one or more pull-strap fastening component formed integrally with one or more fastening components of the main body of the respirator”. A mask with an adjustable and removable strap was also invented for improved mask fit and strap reusability [51]. Steindorf et al. [52] invented a collapse-resistant respirator to tackle the challenge of respirator collapse while breathing. Two fastening components were incorporated to the respirator creating an outward-directed deflection force which helped the main body resist collapse during respiration. Li et al. [53] invented a respirator mask with enhanced breathability by increasing the mask surface area, and Gordon et al. [54] described a respirator with improved fit and air filtration efficiency.

Furuya and Shibata [55] invented a disposable mask with a pair of ears looping that extends from both side of the mask using a nonwoven intermediate layer to block fine particles and able to fit a wide range of individuals with different facial dimensions including children. Another attempted was invented by Mekler et al. [56] using a filtering face mask with two straps and a nosepiece made of flexible semi-rigid material. As claimed, the mask was designed comprising one or more layers to reduce the presence of microbe and dust. The mask layers consisted of nonwoven polypropylene materials with one layer suitable for graphic decoration in an attempt to reduce anxiety and discomfort experience by medical patients as well as clinicians. In another attempt to reduce air leakage from vicinities close to the wearer’s nose and eyes, a disposable non-woven fabric mask was invented with two strings [57]. The folded portion of the mask was adaptable and capable of providing contact with the wearer’s face. In addition, the strings were designed to hold the mask body at a predetermined position on the wearer’s ears or head. Moreover, to form an airtight seal between the edges of a porous filtering media and a wearer’s face, an air-permeable filtering portion was positioned over a wearer’s nose and mouth. An elongated elastic member was anchored to the peripheral bottom portion of the filter to provide an air-inhibiting seal between the air-permeable filtering portion and the wearer’s face [58].

In an attempt to provide a reusable custom fitted surgical facemask with inhalation and exhalation valves, a cup shaped mask body with peripheral edge shaped opening was designed to follow a human’s face contour. Semi-pliable or metal strips were introduced on the interior and exterior surface of the peripheral edges. In addition, a heat-activated thermoplastic member was coupled to the peripheral edges of the mask body [59]. Another face mask was invented including one or more airflow vents at the lower front section and the nasal area. The vents were designed to allow exhalation of heat and CO_2_ and redirect the airflow away from the mask front piece. The mask was designed with an S-shaped filter frame to position the filter material close to the wearer’s nose and mouth [60].

To reduce the risk of viral infection in hospitals, a temperature sensitive surgical mask with layers of thermo-chromatic material that can change color in case of active fever (i.e., temperature > 38 °C) was designed by Eisenbrey and Daecher [61]. In an attempt to develop a comfortable design, a full-face mask with a non-invasive positive pressure ventilation and a continuous positive airway pressure was invented to improve patient compliance and/or treatment [62]. The design was a low-leak mask with an inexpensive and micro-adjustable headgear that allows enhanced sealing and patient’s face fitting [62]. Moreover, another patent proposed a face seal device that corrects the inner face seal leakage and can fit all types of face piece respirators (FFR) used in healthcare institutions and public settlings. The custom fitted face seal was constructed of a heat active thermoplastic copolymer. The face seal was comprised of a geometric design that defined critical fit zones on human facial anatomy which corresponds to known areas of face seal inward leakage [63].

An adjustable face mask was designed with a neck protector and removable semi-soft malleable filter material. This face mask was invented to eliminate fogging of wearer’s goggles by pushing the air down to the sides of the wearer’s face [64]. An earlier face mask design to prevent fogging on wearer’s eyeglass and at the same time facilitate comfort and proper use included a pair of ties that joined to the mask body [65]. The face mask included a sealing member to reduce or eliminate the gaps between the wearer’s face and the upper part of the mask and a barrier panel to reduce or prevent the wearer’s breath from rising towards the wearer’s eyewear [65].

A method for fabricating face masks was proposed by Tai et al. [66] to increase the production rate of fabricating the mask sheets (>120 pieces/min) and the ear loops (>35 pieces/min). The method consisted of advancing continuously a longitudinal nonwoven sheet material that can be divided into a plurality of mask sheets, cutting the sheet material at intervals, and providing a plurality of elastic ear loop strips each of which have two longitudinally opposite strip ends and can be folded to form a pleat between the two strip ends. Moreover, another method of fabricating protective face masks to protect healthcare providers and patients from airborne pathogens was invented by Tsuei [67]. The method tried to overcome the disadvantage of applying separate manufacturing processes of parts that create weak joint points between the front panel and the tie strap. Furthermore, this manufacturing processes is relatively costly and time-consuming, and the joints could be broken and/or create sites of weakness in the facemask. The proposed method provided a continuous web processing, in a specific machine direction, using an elastic nonwoven web and a filtering web to create a unitary structured facemask (i.e., covering wearer’s nose, mouth, and securing the facemask to the head as one piece). Table 1 shows the application and limitation of reviewed patents.

## 4. Filtration Mechanism

The FE of face masks and respirators is the ratio of particles concentration upstream and downstream of the mask. Respiratory droplets are produced by various means such as breathing, talking, coughing, sneezing, and singing. Face mask filtration mechanisms by respiratory droplets and bioaerosols are governed by two major mechanisms: physical mechanisms and electrostatic and thermal rebound mechanisms. Physical filtration mechanisms can be defined as diffusion, interception, impaction, and gravity sedimentation. The filtration mechanism is a function of the particle and fiber size (Reynolds numbers), fiber-based Péclet number (for diffusion), particle-to-fiber size ratio (for interception), and Stoke’s number (for impaction) [68]. Moreover, these mechanisms affect the FE and are a strong function of the particle size and filtration velocity, which yields to the least efficient particle size under a specific range of filtration velocity, namely most-penetrating particle size (MPPS) [69]. In literature, liquid aerosol particles sizes range from 10 nm to 10 µm and are treated as dry solid aerosol particles. This is a reasonable assumption as the particle surface tension is dominant at small scale, and liquid particles behave as solids [8]. Therefore, parameters that affect the FE create nonlinear variation to filtration mechanisms depending on their contribution to the filtration process. On the other hand, electrostatic interaction forces are considered as an essential filtration mechanism especially for enhancing the FE of nano-sized bioaerosols. In addition, nucleocapsid protein crowned *SARS-CoV-2* possesses surface electrostatic potential characteristics [70] that reinforce the importance of the electrostatic interaction role in filtration. In this section, recent research efforts that address the effect of different filtration mechanisms on the FE of face masks and respirators are reviewed.

### 4.1. Gravity Sedimentation

The basis of this mechanism is that large aerosol particles settle due to gravitational forces and do not travel distances more than 1 to 2 m [8,71]. For large particles (>20 µm), gravity is the dominant mechanism [35]; however, it is also proposed that gravity sedimentation and inertial impaction are the main modes of filtration for particles greater than 10 µm [9]. In a case study testing the aerosol FE of common fabrics, it was suggested that ballistic energy or gravity forces were the primary influence on the large exhaled particles ranging from 1 to 10 μm [72]. In addition, it was found that sedimentation, impaction, and interception mechanisms are more important for large aerosol particles within the same range of 1 to 10 μm [6]. Due to the hydrophobicity of medical masks, large particles are not absorbed but rolled down the mask by gravity [8]. It is estimated that 99.9% of the fluid volume consists of large particles and are subjected to gravitational forces and travel only a short distance [73]. Others suggest that particles greater than 5 μm settle due to gravity and can only reach the upper respiratory tract if inhaled [74]. However, a recent study on utilizing cloth face masks to fight the COVID-19 pandemic found that cloth can stop particles smaller than 5 μm, and these particles are filtered by impaction, diffusion, and sedimentation [7]. As a matter of fact, gravitational forces can be completely neglected for particles smaller than 5 μm as they become very small compared to other forces [75,76] and less efficient under large face velocities [77]. In case of viral transmission, large particles either evaporate or break down to smaller sized droplets that can travel for several meters rather than settling due to gravitational forces [70].

### 4.2. Inertial Impaction

As particles’ size, face velocity, and densities increase, the particles’ inertia increases inducing them to change their direction from the airflow streamlines and collide within the filter’s fiber [72,75,77,78]. Both inertial impaction and gravity sedimentation mechanisms are only applicable for medium sized particles (>1 μm to 10 μm) [6,70,79] and on filters made of nonwoven cloth [7,80]. A case study on the commercial Halyard 48207 surgical mask and 3M 1820 procedure masks showed that their FE values were around 80 to 90% and 70 to 80%, respectively, for particles ranging from 0.03 to 0.4 μm, and the efficiency drastically increased to greater than 95% at a particle mobility size of 1.0 μm, for which the filtration mechanism was attributed to interception and inertial impaction [69]. In addition, surgical masks relay on diffusion and inertial impaction for filtration [81]. It has been reported that inertial impaction is the main capturing mechanism for particles larger than approximately 0.3 μm [35,76] to 0.5 μm [72,82]. Moreover, excluding materials that rely on electrostatic interaction as a filtration mechanism, the FE curve has a consistent and typical U-shaped (concave-up) curve, in which inertial impaction and interception increases as particle diameter increases, while diffusion increases as particle diameter decreases. An example of this U-shaped curve was shown by Zangmeister et al. [14] when evaluating the FE for N95 base fabric, surgical masks, and 65%/35% cotton/polyester twill. For a particle mobility diameter of 5 nm, average FE values were found to be 99%, 78%, and 39%, respectively. At 200 nm, the average FE values for all the three filters were lowest and then gradually increased to 89%, 50% and 40% for N95 base fabric, surgical masks and twill, respectively, at a particle mobility diameter of 0.8 µm [14]. Moreover, European standard-face piece respirators, such as FFP2 and FFP3, are designed to capture airborne viruses and rely on the filter’s thickness and its small pore size to provide inertial impaction [83].

### 4.3. Interception

At particle sizes of 0.1 to 1 µm, interception can occur when the particle-filter distance is equal to or less than the particle radius [1,71,80]. Particles follow primary streamlines allowing particle-filter interaction and filtration for particles up to 0.6 µm [72]. However, a recent study suggested that impaction and interception were effective in removing particles larger than 1 µm, while diffusion was more effective at particles smaller than 0.1 µm [79]. When using washable high-efficiency triboelectric air filter, interception and impaction were the main filtering mechanisms at particle sizes of 0.3 µm. The dependence of interception on particle velocity increases as the particle size decreases, but interception also happens when particles do not have adequate inertia to break away from the streamlines. This differentiates interception from inertial impaction mechanisms as particles do not diverge from the airflow streamlines during interception [78]. On the other hand, if the particle size is in the ultrafine or nanoscale, particle-filter collision occurs in a streamline where interception becomes less important than diffusion.

### 4.4. Diffusion

Diffusion is promoted by Brownian Motion of adjunct particles to the filtration media. The particles are deviated from the flow streamline and randomly diffuse through the filtration fabric matrix at particles sizes smaller than 1 µm [1]. Once the particle is collected on the media, another particle comes to the vacant space to be filtered. As the particle size decreases to ultrafine particles (100 nm to 1 µm), the FE becomes more dependent on the filtration velocity, which is governed by diffusion and electrostatic interactions mechanisms [71,84]. In this case, at the highest inhalation flow rate, particle penetration would be the highest (i.e., lowest FE). It is worth mentioning that the effect of Brownian Motion on smaller particles is significant, specifically at particle sizes less than 100 nm [75,76,79] to 200 nm [72]. For instance, it has been shown that diffusion becomes a sufficient mechanism for aerosol particles less than 100 nm filtered by nonwoven fabrics [80], while surgical and any cloth-based masks do not filter by electrostatic interactions but rather employ diffusion and inertial impaction at particle size less than or equal to 1 µm [81]. Diffusion of aerosols particles is usually predicted using the Fickian diffusion model, which assumes that the diffusion flux increases with increased diffusion coefficient (i.e., filter porosity) [85].

Using the classical fibrous theory, the FE results showed that smaller particles were predominated by Brownian Motion [86]. In a study comparing electrostatic charged polyvinylidene fluoride (PVDF) nanofiber filters with diameter sizes ranging from 84 to 525 nm, it was found that electrostatic capturing mechanisms were dominated over diffusion and interception with the expectation of small fiber diameters (84 nm), where diffusion was found to be stronger than the electrostatic mechanism [80]. In addition, diffusion was the dominate mechanism for simulating the coronavirus aerosols at a particle size of 100 nm. As the face velocity and particle size decreased, the particle residence time increased adjacent to the filter media and collision between particles and the filter media increased. Other cases studies showed that as the outflow entered the mask fiber matrix, the particles velocity immediately decreased as they diffused into the mask [72].

### 4.5. Electrostatic Interaction

Nanoparticles at sizes below 0.2 µm are mainly captured by masks that utilize electrostatic interactions as part for their filtration mechanism. However, electrostatic interactions are less affected by particle size rather than flow rates. The filtration is more efficient at low velocities similar to the respiratory velocity due to the allowance of more residence time within the filter’s fabrics [71,72,77]. Electrostatic interaction forces offer effective FE for sub-micron particles without increasing the pressure drop. For filtering facepiece respirators (FFR), such as N99 and N95, ultrafine particles are dependent on the face velocity assuming the lowest collection efficiency at the highest inhalation velocity [84]. Commercially available filters, such as 3M 8210 N95 respirator, Halyard 48,207 surgical mask, and 3M 1820 procedure masks, have electrostatic charges on their fiber surfaces, which increase the FE without compromising the breathability rate [69]. Melt blown polypropylene (PP) non-woven fabrics have relatively large fiber diameters (0.5 to 10 µm) that are wildly used in current masks but are insufficient in capturing particles at sizes smaller than 0.3 µm [1]. On the other hand, PP woven fabrics have lower water adsorption properties than natural fibers or cotton, so PP can retain more static charges [71]. These melt blown PP woven fibers, used in surgical and medical respiratory masks (FFP2 or N95), have surface electrostatic charges and fiber diameters as low as 250 nm that enhance their ability for bioaerosol filtration [9].

**Figure 1:**
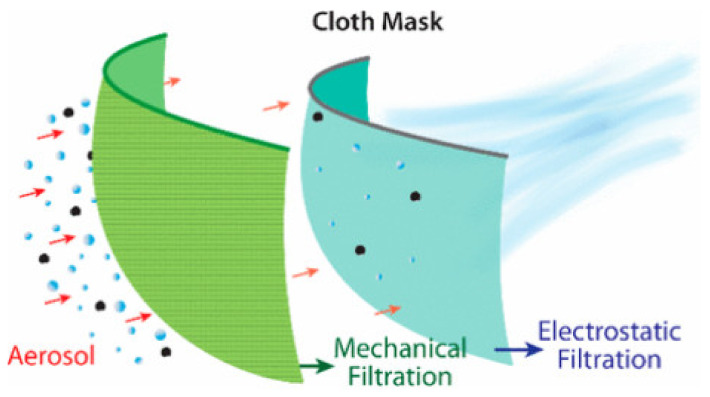
Illustration of filtration mechanism. Reprinted with permission from [71]. Copyright © 2020 American Chemical Society.

A study of 44 samples of household materials and several medical masks using ambient aerosol particles (30 to 100 nm) at low face velocity has found negligible contributions to small particle deposition by electrostatic attraction [77]. FFR is composed of multiple layers with a central layer of synthetic polymer fibers, such as polypropylene, polybutylene terephthalate, and polytetrafluoroethylene, that is electrostatically charged by corona discharge, triboelectrification or electrostatic spinning [87]. For example, N95 masks consist of multiple layers with some electrostatic charges PP layers that significantly contribute to their FE [88]. Therefore, PP, polyethylene, and polyacrylonitrile (PAN) offer sufficient dielectric properties with high electrical resistance and stability for aerosol particle filtration [35]. In addition, for masks fabricated using melt-blowing technique, charges were embraced within the fabric material layers creating a quasi-permanent electric field for adequate filtration. Particles with opposite charges were attracted to these layers by long-rage electrostatic Coulomb force towards the electrocharged layer [81]. As particles get collected on the filter, they remain in place via Van der Waals’ forces [1,89]. Some industrial oils can reduce the electrostatic charge of filters, thus reducing their FE [90]; however, PP and polystyrene (PS) can resist the shielding effect of oil aerosols [81]. The FE of home-made masks made from one layer of tissue paper and two layers of kitchen towels were tested to filter nano sized NaCl aerosols, as depicted in Figure 1.

The viral FE of low-cost non-woven cellulosic fiber filters was studied by fixing poly(ethylenimine) (PEI), and it was found that the non-crosslinked cationic PEI chains created large positive charge density sites available for electrostatic interactions and virus capture [83]. A self-powered electrostatic adsorption face mask (SEA-FM), made of poly (vinylidene fluoride) electrospun nanofiber film (PVDF-ESNF) and a triboelectric nanogenerator (TENG) driven by respiration (R-TENG), was used to filter charged and non-charged coarse particulates (2.5 to 10 μm), fine particulates (1.0 to 2.5 μm), and ultrafine particulates (<1.0 μm) [91]. Results showed that the removal efficiency decreased from 93 to 41 wt% due to the presence of water vapor, accompanied with human respiration, and this affected the filtration performance of the PVDF-ESNF at particulates sizes of 0.5 μm and below. However, the R-TENG supplied electrostatic charges to the PVDF-ESNF, providing SEA-FM the capability to have higher removal efficiencies than commercial masks (i.e., 99.2 wt% removal efficiency for coarse and fine particulates and 86.9 wt% removal efficiency for ultrafine particulates). More recently, a multilayered face mask, comprised of triboelectric series materials (TSM) with an outer layer of metallic mesh comprising electrocution layers (ELs), claimed the ability to filter and deactivate the *SARS-CoV-2* [70].The effectiveness of electrospun fibrous filters in utilizing electrostatic interaction forces for filtration has also been extensively investigated. For example, a manufactured biodegradable electrospun poly(l-lactic acid) (PLLA) fibrous filters achieved a high filtering efficiency of 99.3% for PM2.5 particles. An electret polyethersulfone/barium titanate nanofibrous membrane (PES/BaTiO_3_ NFM) integrated on a nonwoven PP substrate was developed to enhance the filtration performance of airborne particulate matter (PM2.5) through electrostatic adhesion. Results showed that the polarization of BaTiO_3_ nanoparticles (NPs) reinforced charge storage stability on the composite NFMs which enhanced capturing PM2.5 through electrostatic attraction [85].

Four electrospun PVDF nanofibers were electrostatically charged, under optimal conditions to maximize stable charges imparted onto the nanofibers, claimed to capture over 90% of airborne coronaviruses. During filtration, the electrostatic interactions were less effective for smaller size aerosols (<50 nm) due to the smaller dipole moment. Furthermore, filtration tests performed using neutrally charged NaCl aerosols (50 to 500 nm) provided low Coulombic attraction forces compared to tests performed using negatively charged aerosols [80]. The FE of over 15 natural and synthetic fabrics, including natural silk, chiffon (polyester−Spandex), flannel (cotton−polyester), and their combinations, were tested using polydisperse nontoxic NaCl aerosols (10 nm to 10 µm). It was concluded that silk and chiffon were particularly effective at excluding particles (<100 nm) due to electrostatic effects that result in charge transfer within nanoscale aerosol particles [71].

## 5. Filtration Material

**Figure 2:**
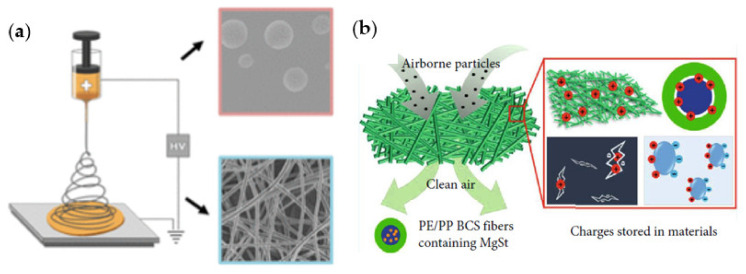
(**a**) Electrospinning process of polymers into fibers; (**b**) schematic of PE/PP nanofiber with magnesium stearate as charge enhancer. Reprinted with permission from [97,98]. Copyright © 2019 American Chemical Society.

Most filter materials are made from a class of materials referred to as ‘nonwovens’ which has minimal airflow resistance and can capture particulates from the air. These nonwovens have web-like structures formed by the entanglement of fibers from polymers such as polypropylene, polyethylene, polyesters, and polyacetonitrile. The web formation step in the production of nonwovens is crucial as each material’s quality depends on the web quality [92]. Some common processes used for web formation include spunbonding, meltblowing, and electrospinning. These processes start with a liquid phase polymer, transformed into fibers and webs in a single step [93]. Spunbonding can produce uniform webs when dealing with high and broadly distributed molecular weight polymers [94]. The meltblowing process involves the formation of super thin, non-continuous fibers, which often have a random arrangement by applying hot air to an extruded polymer melt and drawing it into microfibers [92]. Electrospinning process, as depicted in Figure 2, results in the formation of nanofibers by subjecting a drop of polymer solution to an external electric field [92]. The major difference between these three processes is the size of the fiber produced, which has a strong correlation with web properties such as permeability and mechanical strength. Spunbonding process produces the largest fiber size (15 to 40 µm), melt blowing produces fibers of 2 to 10 µm, while electrospinning produces the smallest fiber size (0.04 to 2 µm) [93]. These small fiber sizes make electrospinning the best process for producing webs with significantly smaller pores, while the thicker fibers from the spunbonding process make it suitable for the production of fibers for mechanical support in the outer and inner layers of respirators [95,96].

The fibrous materials used for mask production (air filtration) should have a high FE and low air resistance. Meltblowing and spunbonding can produce fibers for webs with high FE and low air resistance; however, fibers made via electrospinning have higher electrostatic charges that can yield filters (electret) with higher FEs and lower air resistance [99]. Furthermore, polymer materials used for face mask filtration have low electrical conductivity. Hence, processes like electrostatic spinning and splitting of corona charged film are used during nonwoven production [99].

### 5.1. Common Filter (Nonwoven) Materials

#### 5.1.1. Polyolefins

##### Polypropylene (PP)

Polypropylene is the most common polymer used for producing meltblown and spunbond fibers for making face masks. PP has a relatively low cost and can filter dry particulates. Amongst all synthetic fabrics, PP has the lightest weight due to its low density and specific gravity [94,95]. PP has a high chemical (acid and alkali) resistance and can withstand elevated temperatures up to 150 °C [95]. This material can be reused post decontamination due to its sustained structural integrity. In addition, its smooth surface, ease of processing, recyclability, and micropore distribution uniformity allow PP to be an attractive option for mask production. PP has a modifiable inherent hydrophobicity, good mechanical strength, and abrasion resistance [94].

##### Polyethylene (PE)

This is another common polymer used in meltblown nonwovens. PE is synthesized by polymerizing ethylene monomer. The densities of PE can vary depending on the amount of monomer/comonomer used during the polymerization process leading to the different types of polyethylene; high density (HDPE), low density (LDPE), and linear low-density polyethylene (LLDPE). Like PP, PE has good chemical resistance, light in weight, and is hydrophobic [94,100]. PE is easier to extrude than PP due to the high shear sensitivity and higher melting point of PP resins, resulting in a lower PP yield after extrusion [101]. However, PP is preferred to PE because PP has more mechanical strength and is relatively inexpensive than PE [92].

#### 5.1.2. Polyesters

Polyesters have some advantages over PP such as higher tensile strength, modulus, and heat stability but are not as cost-effective as PP. Another advantage of polyesters is that they can easily be dyed and printed with simple non-aqueous processes. However, it is challenging to recycle polyesters during spunbond manufacturing. Polyethylene Terephthalate (PET) is the most common polyester used in producing nonwoven fibers via spun bonding process [94].

#### 5.1.3. Polyamide

Polyamides, such as nylon 6 and nylon 6-6, have been used for manufacturing of spunbond fabrics. Although nylon has some advantages such as fiber lightness, it also has a high melting point (>260 °C), making it more energy-intensive than polyolefins and polyesters. Nylon fabric readily absorbs water molecules making it unattractive for face mask production even though it can be modified to improve its hydrophobicity [94].

#### 5.1.4. Cellulose Acetate (CA)

CA is an alternative to synthetic polymers since it is derived from biosources, has high FE and hydrophobicity, and is biodegradable. CA selectively filters low level organic compounds, has high water stability, and is soluble in organic solvents [102]. Chattopadhyay et al. investigated the FE of filters made with electrospun CA fibers using aerosolized NaCl particles. It was observed that electrospun CA fibers filters, with much lower thickness, showed a higher FE compared to commercial glass fiber filter [103].

#### 5.1.5. Polylactic Acid (PLA)

PLA is another alternative for synthetic polymers since it is biodegradable and cost-effective. It also has favorable mechanical properties and a smooth appearance. PLA is produced by a polycondensation reaction of lactic acid catalyzed by acid. l-Lactic acid is the common monomer used for this reaction and can be easily produced by lactic fermentation of biowaste by bacteria [104]. Wang et al. [105] fabricated a porous bead on string PLA nanofibrous membrane via electrospinning. It was observed that the morphology of these fibers could largely affect the FE and pressure drop across the membrane. The fiber morphology is affected by the polymer solution viscosity, which is a function of concentration and solvent vapor pressure [105]. A 99.997% FE and a pressure drop of 165.3 Pa were observed in the nanofibrous membrane [105].

#### 5.1.6. Polytetrafluoroethylene (PTFE) Membranes

These are chemically inert membranes that are effective in gas-solid separations. PTFE is widely used as an air filter membrane. It has high filtration performance due to its uniform pore structure with node-connected nanofibrils and low fraction factor. PTFE forms a lightweight and hydrophobic organic membrane with small footprints [106]. These membranes show great chemical stability, high heat resistance, and high surface fracture toughness due to its strong C-C and C-F bonds [107,108]. Biaxial stretching and electrospinning are used to manufacture PTFE nanofibers to achieve a high surface area required to increase contact between particles and fibers while maintaining good particle retention and gas permeability [108]. During the manufacturing process, pore formers such as ZnAc_2_, NaCl, and BaCl_2_ are incorporated to improve air flow [109]. PTFE membranes can be modified for a specific purpose by a wet chemical method, plasma treatment, and irradiation. PTFE membrane surface can be chemically modified without affecting the bulk property using plasma modification. This ranks the technique as one of the most promising surface modification methods [110]. Irradiation using gamma, UV, ion, and electron sources has been shown to change surface property, substrate chemical composition, structure, and morphology of PTFE membranes [111]. Modified PTFE have been shown to have fine particle rejection rate of greater than 99.99% with a pressure drop lower than that of unmodified PTFE membrane [112].

### 5.2. Polymer Composites and Modifications

Nylon 6 is a suitable polymer for face mask production due to its strong affinity for particulate matter and sufficient air permeability; however, masks made with this material can have significant thermal discomfort, especially in temperate regions [113]. This thermal discomfort which depends on the thickness of the mask material, is challenging to adjust since thickness also correlates with particle matter removal for nylon fibers and nanoporous PE. Yang et al. [113] demonstrated the enhancement of thermal comfort in a novel face mask made of nylon 6 nanofibers on nanoporous PE. In addition, nanoporous PE was used as a co-substrate because of its transparency to mid-infrared radiation emitted by the human body. The fiber/nano PE showed adequate cooling properties, low-pressure drop, and FE (~99.6%) at high temperatures. Further studies showed that a layer of silver could be used to modify the nano PE substrate to reflect the radiation from the human body leading the warmth in colder regions [113].

Water resistance is an essential feature of a good face mask material. PTFE has been the common polymer for making waterproof membrane filters, but its high cost and difficulties in regulating the porous structures have led to further research on better alternatives. Polyurethane, polyacrylonitrile, and polypropylene have been used as alternatives, but these polymers have inadequate hydrostatic pressure. Amini et al. [114] developed a waterproof breathable membrane for face masks using a combination of polyvinylidene fluoride (PVDF) electrospun membrane and a hydrogel electrospun mat which could be a better alternative to PTFE. This was achieved by subsequently electrospinning a layer of hydrogel on a PVDF electrospun mat. The hydrogel comprised of polyvinyl alcohol (PVA) and polyacrylic acid (PAA) fused by an esterification reaction. The hybrid membrane showed an improved water vapor permeability (WVP) with good water resistance and windproof property [114].

Akduman et al. [102] used CA and PVDF nanofibers as layers for N95 respirators and compared the test results to the National Institute for Occupational Safety & Health (NIOSH) standards. Smooth nano fibers of both polymers were obtained via electrospinning. The authors observed that 16% (*w/v*) and 15% (*w/v*) CA, collected at 60 and 30 min (16CA60 and 15CA30) respectively, met the NIOSH airflow requirements and could be used for N95 production. They reported that fiber thickness had a significant effect on filtration performance, and the thickness had a close correlation to the polymer concentration. The results also showed that the NIOSH requirement for the N95 particulate filtering half mask of at least 5% penetration and Δ*P* of 35 mmH_2_O could be achieved using these nanofibers. The high FE of (16CA60 and 15CA30) of CA nanofibers was attributed to the fiber bulkiness, which supports surface filtration, interception, and diffusive effects. PVDF produced thinner nanofibers and was reported to meet NIOSH requirements at concentrations where double-layered face-to-face nanofiber mats were made with 10% (*w/v*) PVDF [102].

Composite nanofibers of polyacrylonitrile (PAN) and graphene oxide (GO) have also been considered for use as membrane filters for face masks. Li et al. [115] attempted to modify PAN filters using GO to obtain a very porous membrane structure, resulting in pressure drop reduction. The composite (GOPAN) showed a relatively narrow pore size distribution range between 0.5 to 2.5 µm, confirming homogeneous pores in the membranes. The 0.5 mg GO with 1 g PAN (05GOPAN) nanofibers effectively impeded the diffusion of smoke, confirming its ability to hinder diffusion of tiny particles. It was observed that the composite filter had a higher FE (99.97%) and lower pressure drop (8 Pa) compared to pure PAN (93.36%, 22 Pa) or other GOPAN concentrations [115]. The composite 05GOPAN was tested for use as a membrane filter in a surgical mask. Contrary to non-woven filter materials, the composite filter was observed to absorb more contaminants with wearing time.

Liu et al. [116] added low melting polyethylene oxide (PEO) to a composite membrane which comprised of PSF and PAA by binding in-situ, forming physical bonding structures between the fibers and giving the resulting membrane an anti-deforming property. The good mechanical properties, high FE of about 99.992%, low pressure drop (95 Pa), and a high-quality factor of the resulting composite makes it a promising candidate for respirator production [116].

Nanofibers from PP and PE composites have high mechanical strength and chemical resistance, low air resistance, low moisture absorption with high heat resistance, and excellent electrical insulation compared to their individual pure counterparts [117]. Due to the electret property of the composite nanofiber, it can be charged to increase FE; however, charges can escape leading to a decrease in the FE. To improve charge stability, Lui et al. made a PP/PE bicomponent filtration material with magnesium stearate particles, a nucleating agent [98]. The results obtained over 90 days using this novel material showed a lower reduction in FE (98.94 to 94.9%) compared to conventional PE/PP membranes (93.92 to 86.06%). This confirmed an improved surface potential and charge storage stability. The enhanced charge stability was attributed to a change in the crystalline structure of the bicomponent polymer caused by the nucleating agent [98].

## 6. Filtration Experiments and Testing Practices in Academic Research

**Figure 3:**
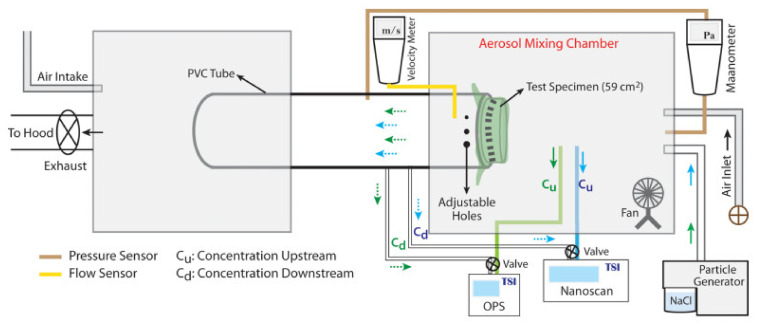
Illustration of filtration efficiency test setup using aerosolized NaCl. Reprinted with permission from [71]. Copyright © 2020 American Chemical Society.

Since the COVID-19 outbreak, much research has been conducted aiming to reduce the cost of face masks and respirators as well as improving their FE, especially, against bioaerosol particles. These studies include, but are not limited to, developing advanced filtration materials, testing publicly used and non-certified filtration materials during the pandemic, designing new mask configurations with high efficacy for public use, and providing a better understanding of different filtration mechanisms. Depending on the research hypothesis and objective(s), these research efforts have used different testing methods and practices to model/stimulate real environmental conditions (i.e., type of aerosol particles, aerosol generation and loading, and real time FE evaluation under different scenarios). Under time-limited research investigation periods and difficulties in experimentally simulating specific parameters, experiments were performed to the best practice and available resources. However, each research study had its own experimental condition(s) and testing environment(s) to reduce and control its parametric uncertainties. An example of an experimental setup to measure the FE of aerosolized NaCl is shown in Figure 3. In this section, recent experimental testing methods and practices, conducted in the past five years, are summarized with specific focus on research studies published during the COVID-19 pandemic. Table 2, Table 3, Table 4, Table 5, Table 6, Table 7, Table 8 and Table 9 specify different research investigations including their objective(s), tested mask material(s), modeled aerosol particle(s), particle generators and their experimental setup, and highlights on FE outcomes using different modeled aerosol particles. It must be noted that this table does not include case studies that focused on regeneration and decontamination of face masks, as this will be discussed in Section 7.

## 7. Current Practices of Decontamination and Regeneration of Face Masks and Respirators

**Figure 4:**
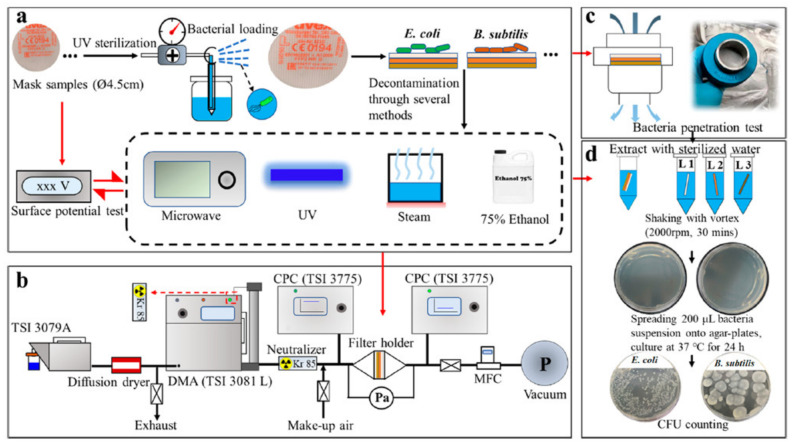
A schematic showing (**a**) various setups for decontamination and regeneration, (**b**) setup for particle filtration efficiency test post decontamination, (**c**) bacteria penetration test setup, and (**d**) colony unit counting post bacteria filtration. Reprinted with permission from [136]. Copyright © 2020 American Chemical Society.

Demand for face masks and respirators can increase significantly during a pandemic, and it is very vital to maintain a steady supply to ensure the safety of all individuals. Treatment and reuse of face masks can reduce the load on supply chains and reduce the environmental pollution caused by single-use masks disposal. As shown in Figure 4, several methods have been used for decontamination, such as thermal disinfection (dry or wet), mild chemicals, microwave, ultraviolet light, and detergents.

### 7.1. Thermal Disinfection

Heat treatment methods for mask decontamination are more suitable for the decontamination of masks at home due to availability of heating systems. The effect of heat on mask decontamination can be affected by temperature and relative humidity. Campos et al. [137] investigated the effect of heat on pathogens for face masks treatment at different relative humidity. N95 grade surgical type masks were decontaminated from three viruses, *SARS-CoV-2*, Human coronavirus *NL63* (*Hcov-NL63*), and *chikungunya virus*, at temperatures above 85 °C and at 100% relative humidity, and results showed no viruses were detected on the masks’ surfaces after 20 min. Treatment performed at 85 °C and 60% relative humidity for 20 min showed a 4.3-log 10 reduction compared to 5.02-log 10 reduction obtained at 100% relative humidity. Filtration performance was unaffected after 20 cycles between the temperature range of 75 to 85 °C for 20 to 30 min/cycle, respectively, at a relative humidity of 100% [137]. A conventional electric cooker was used to decontaminate respirators infected with *rotavirus* (RV), *adenovirus* (AdV), *Tulane virus* (TV), *human virus type 2*, and *porcine transmissible gastroenteritis virus* (TGEV) at 100 °C and a relative humidity of 5% for 50 min [138]. It was found that the respirator integrity, which included the filtration performance and fit of the respirator, was unaffected after 20 cycles of this treatment. Under the same treatment conditions, there was a greater than 5.2-log10 reduction in viral activity for TV, 6.6-log10 for RV, 4.0-log10 for AdV, and 4.7-log10 for TGEV, which were all below the detectable limits of the viruses [138]. CY Seun et al. [139] reported using an oven at 100 °C for 15 min, a steam cooker at 100 °C for 10 min, a water bath at 100 °C for 10 min, and an autoclave at 121 °C for 20 min to decontaminate *S. aureus* contaminated surgical mask. A decrease in FE was observed for all treatments except dry heating, by oven, which did not show a significant decline in FE after three cycles (i.e., mask samples maintained about 95% FE). All heating methods showed complete deactivation of bacterial activity of *S. aureus* up to a greater than 4-log10 reduction. It was also reported that dry heat did not show significant effect on mask hydrophobicity; however, there were structural changes in mask materials after boiling, steaming, and autoclaving [139]. Steam at a temperature less than 100 °C and normal atmospheric pressure was used for bacterial deactivation on a particle filtering and surgical mask surface contaminated with *Escherichia coli* and *Bacillus subtilis* [136]. A 100% deactivation was observed after 90 min of treatment; however, a slight decay in electrostatic property, which affected mask FE, was also observed [136]. Kumar et al. [140] autoclaved various models of N95 respirators contaminated with either *Vesicular stomatitis virus (VSV)*, *Indiana serotype* or *SARS-CoV-2,* at 121 °C for 15 min for disinfection. Results found no significant changes in functional integrity for all mask samples after ten cycles, along with greater than 6-log10 reduction of infectious virus was reported post treatment [140]. Begail et al. [141] used dry heat at 102 °C for 60 min to disinfect respirators contaminated with *porcine respiratory coronavirus* (PRCV), and reported a viral infectivity reduction by two orders of magnitudes. Lastly, Daeschler et al. [142] used heat at 70 °C and relative humidity ranging from 0 to 70% for about 60 min to decontaminate *SARS-CoV-2* and *Escherichia coli* infected face respirators. Post treated respirators showed greater than 95% of FE after ten cycles, while no infectious viruses were detected after dry heating at 70 °C for 60 min. In addition, *E. coli* was deactivated after heating at 70 °C for 60 min and at a 50% relative humidity [142].

### 7.2. Microwave

The use of microwave radiation and generated steam is also a non-chemical form of decontamination, and it is particularly promising because of its potential for home use. He et al. [136] used a 400 W microwave for 10 min to disinfect *Escherichia coli* and *B. subtilis* contaminated particle filtering and surgical mask face mask, and reported deactivation of *E. coli* and *B. subtilis* to be above 98% or greater than 4-log reduction; however, mask morphology was affected over a long period of microwaving. The effect of a higher power microwave (1100 W) on facepiece respirator FE was evaluated after a 2 min exposure with 1 min on each side of the mask [143]. No significant drop in particle FE was observed after a 2-min exposure when using polydisperse sodium chloride aerosol for the test; however, N95 grade filters melted after four minutes, forming visible holes [144]. Bergman et al. [143] reported deformation of the mask samples’ head straps along with separation of mask cushion after treatment with a 1100 W microwave for 2 min. Lastly, Jung et al. [145] investigated the effect of a microwave of 750 W power for 1 min on respirator FE, and found insignificant impacts on respirator FE when tested with sodium chloride aerosol particles.

### 7.3. Ultraviolet Irradiation (UVI)

Short-wave ultraviolet (UV) light has been used as a disinfectant for more than a century since UV light kills or inactivates microorganisms by disrupting their DNA and replication. However, UV cannot inactivate a virus or bacterium if it is covered by dust or soil, embedded in porous surface, or on the underside of a surface; that is, inactivation only occurs if microorganisms are directly exposed to UV lights. The effect of UV on mask decontamination was reported using a 5.5 W UV lamp for two or more minutes to decontaminate respirators infected with *Porcine Respiratory Coronavirus* (PRCV), which resulted in a significant decrease of virus infectivity by three orders of magnitude post decontamination [141]. He et al. [136] used a 254 nm wavelength at 126 mj/cm^2^ for five minutes to decontaminate *E. coli* and *B. subtilis* from face masks. An insignificant effect was observed on mask FE, and the treatment resulted in a 100% deactivation of *E. coli*. Furthermore, the combination of UV light and microwave to decontaminate bacteria-infected face masks showed a 100% deactivation of *E. coli* and *B. subtilis* in a 5 min UV followed by a 4 to 12 min microwave treatment without impacting the mask structure [136]. Viscusi et al. [144] investigated the effect of respirator FE using a 40 W UV light with intensity between 0.18 to 0.20 mW/cm^2^ for 30 min (15-min exposure on each side), and observed no significant drop in particle FE after testing with polydisperse sodium chloride aerosol. Jung et al. [145] reported that a 10 W UV lamp could be used to disinfect respirators with an 82% deactivation of the *E. coli* after a 1 h exposure of both sides of the respirator without a significant impact on the FE. Lastly, Lindsley et al. [146] reported a slight decrease in FE (up to 1.25%) after treating respirators with a 950 j/cm^2^ UV light.

### 7.4. Chemicals

Chemicals have been widely used for sterilization purposes and have also been attempted for masks decontamination. Some alcohols and peroxides have shown significant effect on pathogen deactivation; however, the negative effect of these chemicals on filter electrostatic property should be considered when choosing a chemical treatment. Jatta et al. [147] used 59% vaporized hydrogen peroxide (VHP) to disinfect two models of N95 respirators, 3M 8211, and 3M 9210, without any significant drop in FE after ten cycles. Likewise, Begail et al. [141] used 59% VHP with a peak VHP concentration of 750 ppm to decontaminate PRCV and determined that the virus infectivity was reduced by one order of magnitude. He et al. [136] treated bacteria-infected respirators using 75% ethanol for two minutes. While a significant effect on the mask surface potential and change in mask morphology were reported, bacteria were completely deactivated. Kumar et al. [140] investigated respirator decontamination from *Vesicular stomatitis virus*, *Indiana serotype* (VSV), or *SARS-CoV-2* using ethylene oxide for 60 min, low-temperature hydrogen peroxide gas plasma (LT-HPGT) for 47 min, VHP with peak VHP concentration of 750 ppm, and peracetic acid fogging (PAF). It was observed that ethylene oxide maintained mask FE after three cycles for all mask samples tested. LT-HPGT treated masks lost some FE after the first cycle, while VHP and PAF treatments maintained both functional and structural integrity after ten cycles. There was a greater than 6 log 10 reduction of infectious virus for all methods [140]. Jung et al. [145] used several solutions, such as 5.5% sodium hypochlorite (NaClO), 70% (*v/v*) ethanol solution, and 100% isopropanol each used for 10 min to decontaminate face respirators contaminated with *E. coli*. The solution of NaClO and NaOH had no significant adverse effect on FE, unlike the ethanol and isopropanol solutions, which showed a 28% decrease in FE. All solutions resulted in a 100% removal of bacterial from the mask surface [145]. Suen et al. [139] attempted the use of 0.55 (w/v) of Ultra axion, a household detergent, solution in deionized (DI) water for 30 min to decontaminate a face mask contaminated with *S. aureus*. The solution did not successfully deactivate *S. aureus* and significantly decreased the FE after the first cycle. Jung et al. [145] investigated the impact of laundry, with and without detergent, on face respirator filter efficiency. The respirator sample (N95 grade) was agitated for 10 min at 90 rpm and 24 °C in water alone or with added 0.1 wt% of detergent. No significant change in FE was observed after decontamination with water alone, but there was a substantial decrease in FE after decontaminating with detergent [145].

Other methods of decontamination and regeneration have been attempted in laboratories and are currently under improvements like the use of nanoparticles. Li et al. [148] investigated the effect of coating a surgical face mask surface with silver nanoparticles on *E. coli* and *S. aureus* and reported a 100% deactivation of both bacteria in the presence of silver nanoparticles after 48 h of incubation. This was attributed to the distortion of bacterial cells’ morphology leading to damage in the bacteria enzyme. It was also reported that nanoparticles did not result in skin irritation when the mask was worn [148]. Table 10, Table 11, Table 12, Table 13 and Table 14 show the decontamination and regeneration investigations that have been recently conducted on face masks and respirators using thermal disinfection, microwaving, ultraviolet irradiation, chemicals, and laundry detergent, respectively.

## 8. Conclusions

The review presented here highlights the recent efforts in developing face masks and respirators to prevent the transmission of bacterial and viral respiratory tract infections among healthcare works, patients, and general public. Limitations on mass production in manufacturing along with the inability to supply affordable and efficient face masks to meet public demand were observed during the pandemic. Face mask manufacturing was further hindered by the public’s ability to produce homemade masks with varying levels of protection and filtration capabilities. Several factors have limited the production of face masks during the first wave of the pandemic including lack of scientific data, inability to meet demand at affordable costs, and presence of uncertainty parameters in predicting FEs under different environmental conditions. In addition, the discomfort associated with face mask wearing along with the impact of mask fit on FE results decreased their popularity. Alongside comfort and fit issues, the options for filtration material selection are controlled by the ability to provide adequate breathability rates and minimal pressure drop across the filter without compromising the filtration capability. Adding to the above complexity associated with face mask design and FE evaluation, the selection of the mask is also dependable on the application and the practical length of mask usage. For instance, prolonged and/or continuous use of face masks, in some cases, may lead to negative effects including headaches, rash, and skin breakdown among others.

To obtain comparable results from case studies, research testing procedures should be standardized. For example, particle shape, morphology, and concentration impact the FE. Therefore, improving particle generation procedures as well as generation testing equipment and instrumentation, in a standard experimental setup, have the capability of providing a better understanding of the filtration mechanism(s) across different filtration materials. In addition, it would provide more confident air filtration scenarios due to different human activities such as speaking, breathing, coughing, and sneezing. It is also recommended that future studies consider applying test conditions and protocols that are approved, or at least acceptable, by industry standards.

Polymeric materials used for face mask production are usually non-biodegradable, which can lead to environmental concerns as most countries have poor recycling practices. In recent times, biodegradable options have been explored; however, more work is required in replacing synthetic polymers with a cost effective non-synthetic material. Electrospinning has shown promise in making quality fibers of polymers from natural sources which can make face masks with high FEs and low air flow resistance. Table 15 shows the different types of face masks and the most common materials used for each type.

Decontamination and regeneration can be used to make face masks readily available during shortages, as experienced during the initial phase of the *SARS-CoV-2* pandemic, while reducing the polymer waste in the environment. These regeneration techniques are usually cost effective; however, they can have negative impacts on FEs and the overall quality of the mask material. Moreover, collating and shipment of contaminated face masks to decontamination sites can be labor intensive and pose health risks. More extensive research is recommended for developing face masks (mask materials) with microbial deactivation or growth impeding properties to ensure safe reuse, hence, reducing shortages and maintaining a safe environment.

As challenging as it has been, the pandemic surge has highlighted the urge to involve multidisciplinary parties to solve the common global goal of developing, testing, and manufacturing protective and affordable face masks. International trading, globalization, and economic interdependence along with advancements in communication and transportation were enough factors to spread the contagious coronavirus and cause a pandemic in early 2020. A global solution requires collaborative application of science, engineering, policy, and public affairs in order to develop publicly affordable face masks that can meet compliance and reduce the transmission risk of the coronavirus, its variants, and other possible contagious viruses in the future. It must be understood that different types of masks have their own advantages, disadvantages, capabilities, and limitations. However, there could be ideal and universal masks for each specific application that can assure global public safety.

## Figures and Tables

**Table 1:** Applications and limitations of reviewed patents.

Patent No: Title	Applications	Limitations	Reference
US5553608A: Face mask with enhanced seal and method.	For preventing passage of fluids between mask periphery and wearer	Fogging of glasswear	[38]
US5704349A: Surgical face mask with darkened glare-reducing strip and visor	Prevents movement of fluids from mask exterior to wearers face	May not be comfortable to wear	[39]
US3888246A: Anti-fog surgical face mask	Reduces fogging of wearer glasses(goggles)	No mention of proper face fitting	[43]
US6988500B1: Fog free medical face mask	Facilitates escape of exhaled moist air reducing fogging of wearers glass	No filtration efficiency test	[44]
US9770611B2: Maintenance-free anti-fog respirator	Prevents fogging by altering intrinsic structure of the mask sinus region	No filtration efficiency test	[45]
US10357672B2: Apparatus, system, and method to prevent fogging of eyewear.	Fogging is decreased by removal of exhaled air through the side vents	May have gaps between mask and wearer’s nose	[46]
EP1479413A2: Face mask that has a filtered exhalation valve	Efficiently allows movement of exhaled air	Fogging of wearers glasses may not have been considered	[47]
US6928657B2: Face mask having hook and loop type fastener	Better fitted mask to reduce wearers exposure	May not have antifogging abilities	[48]
US20090044812A1: Vent and strap fastening system for a disposable respirator providing improved donning	Uses exhalation vent to prevent fogging and has comfortable straps	No filtration efficiency data	[50]
US20110180078A1: Face Mask with Adjustable and Detachable Straps	Improved mask fit with detachable and reusable straps	Could have fogging problems	[51]
US8267088B2: Collapse resistant respirator	Allows for breathing without mask collapse	No pressure drop data	[52]
EP3653266A1: Respirator Mask	Increased surface area for enhanced breathability	No pressure drop test to confirm enhanced breathability	[53]
US10799728B2: Respirators and related methods	Improved fit and filtration efficiency	No apparent limitation	[54]
US20210022418A1: Disposable mask	Improved fit for individuals of different ages and various facial dimensions	Could have gaps between mask and wearer’s face	[55]
US9655392B2: Filtering face mask	Effective facemask with provision for graphical design	Fogging of wearer’s glass	[56]
US9386813B2: Mask	Provides airtight seal between face mask and wearer’s face preventing inhalation of contaminated air	Could fog glassware since only the side of the mask is airtight sealed	[57]
US20160151650A1: Fitted face mask	Inhibits wearers exposure to unfiltered air	No pressure drop data to show breathability	[58]
US9320923B2: Surgical face mask, including reusable masks, with filtered inhalation and exhalation valves	Replaceable valves and reusable straps	No pressure drop test	[59]
US9457207B2: Facemask with filter insert for protection against airborne pathogens	Equipped with vents which allows exhalation of heat and CO_2_	No apparent limitation	[60]
US20190125011A1: Temperature sensitive surgical face mask for identifying at risk patients and reducing viral infection	Made up of a thermo-chromatic material which changes color with temperature	May not prevent fogging of wearers glass	[61]
US10307554B2: Mask and components thereof.	Improves mask comfort of the wearer	No pressure drop data to show breathability	[62]
US20170095681A1: Adjustable facial conforming face mask	Reduces fogging of wearer’s goggles by channeling exhaled air to the side of the mask	Maybe bulky and uncomfortable to wearer	[63]
US20160235136A1: Surgical mask	Eliminates the gap between the wearer and the mask by incorporating a sealing member	No pressure drop test	[65]
US9706800B2: Face mask and method for making the same	Developed a method for face mask production for increased production rate	N/A	[66]

**Table 2:** Summary of Face Mask and Respirator Filtration Experiments and Testing Practices Using NaCl Particles.

Purpose of Study	Mask Material	Modeled Aerosol Particles	Experimental Aerosol Particle Generator	Notes on Filtration Efficiency	Ref.
Applied a particle counting approach, and additionally pressure drops (*Dp*) were determined for 44 samples of household materials and several medical mask Performed systematic measurements of particle size-resolved (30 nm to 10 µm) FE and of pressure drop for 44 typical household materials and several medical masks under different experimental conditions, including different face velocities, number of sample layers, and leaks	48 different sample materials	Charged and neutralized NaCl aerosol. All measurements were performed with particles of 30 nm, 50 nm, 100 nm, 250 nm, and 500 nm diameter	Generated NaCl aerosol by: ➢ Nebulizer (model 3076, TSI, Inc.) ➢ Silica gel diffusion dryer A differential mobility analyzer (DMA, model 3081, with X-ray aerosol neutralizer model 3088, both TSI, Inc.) was used to generate monodisperse aerosol of the desired *dp.*	FE and pressure drop measured for different numbers of material layers showed that each layer can be treated as individual filter Samples with defined leaks covering (0.5% to 2%) showed total FE reduction by 50% to two thirds of the value obtained with leak-free samples Larger FE found for large particles (*dp* > 2.5 mm) compared to small ones (*dp* < 100 nm) With increasing face velocity, a decrease in FE for small particles (*dp* ≤ 250 nm) and an increase in FE for large particles (*dp* ≥ 2.5 µm)	[77]
Evaluated FEs as a function of aerosol particulate sizes in the 10 nm to 10 μm range, which is particularly relevant for respiratory virus transmission	Several common fabrics including N95, surgical masks, cotton, silk, chiffon, flannel, various synthetics, and their combinations	A polydisperse, nontoxic NaCl aerosol *dp* (10 nm to 10 μm)	Generated aerosol particles by (NaCl) aerosol generator (TSI Particle Generator, model #8026)	FE single layer ➢ 5% to 80% (*dp* <300 nm) ➢ 5% to 95% (*dp*>300 nm) FE improved when multiple layers were used and when using a specific combination of different fabrics. FE of cotton/silk, cotton/chiffon, cotton/flannel: ➢ >80% (*dp* < 300 nm) ➢ >90% (*dp* > 300 nm)	[71]
Developed a highly breathable and thermal comfort filter medium consisting of electret polyethersulfone/barium titanate nanofibrous membrane (PES/BaTiO_3_ NFM) integrated on a nonwoven polypropylene substrate	Electret PES/BaTiO_3_ NFM PES (E3010) was purchased from BASF Co., Ltd., Germany Barium titanate NPs (BaTiO_3_, 20–60 nm) was supplied by Shanghai Aladdin Chemical Co., China *N,N* dimethylformamide (DMF) and *N*-methyl-2-pyrrolidone (NMP) were obtained from Shanghai Chemical Reagents Co., Ltd., China	NaCl aerosol particles (PM2.5)	The air permeability was tested according to ISO 9237 (1995) standard test method using a Frazier Air Permeability Tester (YG461E, NBFY Co. Ltd., China) with a fixed testing area of 20 cm^2^ and the pressure drop of 200 Pa	The electret PES/BaTiO3 NFM1.5 medium with a low basis weight of 4.32 g/m^2^: ➢ FE (99.99%) ➢ Pressure drop of 67 Pa after being treated at 200 °C for 45 min	[85]
Presented practical design principles for the fabrication of electrocharged filtration layers employed in N95 FRs using commonly available materials and easily replicable methods	Four N95 FR Four taped and non-taped surgical masks Four polypropylene surgical masks Four polypropylene 3D design masks Four polypropylene-polystyrene surgical masks Four polypropylene-polystyrene 3D design masks	Charged and uncharged NaCl aerosol particles PM2.5 For Nanofiltration test: polystyrene nanospheres fluorescently tagged with Dragon Green (480 nm Absorption wavelength, 520 nm Emission wavelength) with mean diameter 50 nm ± 10 nm (Manufacturer Bangs Labs, Catalog No. FSDG001, Lot No. 14092) were used as a substitute for the *SARS-CoV-2* virion	Mist generator Inexpensive piezoelectric atomizer (APGTEK Aluminum Mist Maker) usually employed in home decoration was submerged in sodium chloride solution (5% by weight NaCl in de-ionized water) to generate aerosol particles The generated mist was exposed to negative ion air purifier to charge the aerosol particles for some of the tests For Nanofiltration test: the aqueous solution was first dried and the nanoparticle dust was exposed to a brief burst of coronal discharge	Penetration percentage range values measured from filtration tests: ➢ N95 FR (1.43% to 3.37%) ➢ Taped and non-taped surgical masks (11.98% to 47.18%) ➢ Polypropylene surgical masks (7.3% to 12.26%) ➢ Polypropylene 3D design masks (3.62% to 5.14%) ➢ Polypropylene-polystyrene surgical masks (6.22% to 12.31%) ➢ Polypropylene-polystyrene 3D design masks (2.97% to 3.53%)	[81]
FE, differential pressure (Δ*P*), quality factor (QF), and construction parameters were measured for 32 cloth materials (14 cotton, 1 wool, 9 synthetics, 4 synthetic blends, and 4 synthetic/cotton blends) used in cloth masks intended for protection from the *SARS-CoV-2* virus (diameter 100 ± 10 nm)	Seven polypropylene-based fiber filter materials were also measured including surgical masks and N95 respirators. Cotton Synthetic Synthetic blend Synthetic/cotton blend Paper Polypropylene-based	NaCl aerosol with particle mobility diameters *dp* (50 to 825 nm)	Aerosol was generated from a 10 mg/mL aqueous solution of NaCl using a constant output atomizer supplied with dry (dew point < −75 °C), HEPA-filtered air (25 psig)	The FE and Δ*P* increased monotonically with the number of cloth layers for a lightweight flannel, suggesting that multilayered cloth masks may offer increased protection from nanometer-sized aerosol with a maximum FE dictated by breathability (i.e., Δ*P*) The measured data indicate that particle charge does not impact FE for both natural and synthetic fabrics.	[14]
Assessed the fitted filtration efficiencies (FFEs) for face mask alternatives used during the COVID-19 pandemic	3M 1860 N95 Respirator Surgical mask with ties Procedure mask with ear loops	NaCl particles Median *dp* (0.05 μm)	A particle generator 8026 (TSI) was used to supplement ambient particle counts in the chamber	FFE using the Occupational Safety and Health Administration Modified Ambient Aerosol CNC Quantitative Fit Testing Protocol for Filtering Facepiece: ➢ 3M 1860 N95 Respirator (98.5 ± 0.4%) ➢ Surgical mask with ties (71.5 ± 5.5%) ➢ Procedure mask with ear loops (38.1 ± 11.4%)	[2]
Comprehensively evaluated the overall and size-dependent filtration performances of non-medical materials	Total number of 43 combinations: ➢ Four types of medical materials ➢ Thirteen types of non-medical materials	NaCl aerosols *dp* (20 to 550 nm)	Generated by a constant output atomizer (Model 3076, TSI Inc.) nebulizing a NaCl-water solution with a mass concentration of 0.1%	Fibrous filters, such as household air filters, can achieve a FE and flow resistance similar to that of N95 mask materials Fabrics, such as a scarf, bandana, and pillowcases with different thread counts, are relatively inefficient for collecting aerosols while inducing a large pressure drop, which may lead to difficulty in breathing A positive relationship between the thread count of the fabrics and the FEss was observed	[79]
Aimed to investigate the FE of home-made masks that could be used as alternatives for community mitigation of COVID-19	Home-made masks Medical masks (as the control)	NaCl aerosols *dp* (6 to 220 nm)	Scaled air duct system by using nebulizer	The FE of the home-made masks at 6 to 200 nm were non-inferior to that of medical masks (84.54% vs. 86.94%, *p* = 0.102) Both types of masks achieved an FE of 90% at 6 to 89 nm A significantly higher FE was achieved when one piece of tissue paper was added adjacent to the inner surface of the medical mask than medical mask alone: ➢ (6 to 200 nm: 91.64% vs. 84.75%, *p* < 0.0001) ➢ (6 to 89 nm: 94.27% vs. 90.54%, *p* < 0.0001) ➢ (90 to 200 nm: 82.69% vs. 73.81%, *p* < 0.0001)	[86]
Evaluated the filtration properties of natural and synthetic materials using a modified procedure for N95 respirator approval	Common fabrics: ➢ Polypropylene (particulate FFR and medical face mask) Cotton (T-shirt and sweater) Polyester (toddler wrap) Silk (napkin) Nylon (exercise pants) Cellulose (paper towel, tissue paper, and copy paper)	Polydisperse NaCl aerosols *dp* median (0.075 ± 0.02 μm)	All tests were conducted on an Automated Filter Tester 8130A (TSI, Inc.) A modified version of the NIOSH standard test procedure was used to measure the FE and pressure drop of tested materials	FE values: ➢ Common fabrics of cotton, polyester, nylon, and silk (5% to 25%) ➢ Polypropylene (6% to 10%) ➢ Paper-based products (10% to 20%) An advantage of polypropylene spunbond is that it can be simply triboelectrically charged to enhance the filtration efficiency (from 6 to >10%), without any increase in pressure (stable overnight and in humid environments)	[74]
Demonstrated the low-cost (<$300) conversion of standard equipment used to fit-test respirators in hospital and industrial settings into a setup that measures quantitative FEs of materials based on NIOSH N95 guidelines, and subsequently measure FEs of materials found in healthcare and consumer spaces	Sample materials available in the hospital (healthcare-grade) and those accessible to the public (consumer-grade)	NaCl particles *dp* (0.02 to 1.0 µm)	A particle generator (TSI 8026 Shoreview, MN) generated aerosolized NaCl particles with a median diameter of 0.04 microns and geometric standard deviation (GSD) of 2.2 in a chamber (plastic storage bin) to provide a steady-state supply of particles at sufficient concentration (>4000 particles/cm^3^)	FE values: ➢ A double-layer of sterilization wrap used in surgical suites (96.49%), pressure drop of 25.4 mmH20 ➢ 3M 1870 (99.43%) ➢ 1860 N95 respirators (98.89%) ➢ Consumer grade materials (35% to 53%) ➢ A combination of consumer-grade materials (90.37%)	[118]
Examined the ability of fabrics that might be used to create home-made face masks to filter out ultrafine (0.02 to 0.1 μm) particles at the velocity of adult human coughing	Several common fabrics and their combinations.	Aerosol ultrafine particles *dp* (0.02 and 0.1 μm)	Generated aerosol particles by nebulizing NaCl with a nebulizer (Pari Pro Plus, Vios, USA, 312F83-LC+) at the total output rate of 590 mg/min	Average FE: ➢ Single layer fabrics (35%) ➢ Layered combination (45%) Non-woven fusible interfacing, when combined with other fabrics, could add up to 11% additional FE Fabric and fabric combinations were more difficult to breathe through than N95 masks	[12]
Addressed the development of a novel charged PVDF nanofiber filter technology to effectively capture the fast spreading, deadly airborne coronavirus, especially COVID-19, with targeted aerosol size set at 100 nm (nano-aerosol), and not 300 nm	PVDF nanofiber filter average fiber diameters 84, 191, 349 and 525 nm	Neutrally charged sodium chloride aerosols *dp* (50–500 nm)	Sub-micron aerosol generator	Two filters, with low basis weight (<1 gsm fibers), could meet the 90% efficiency target for 100-nm aerosol with pressure drop less than 30 Pa: ➢ 3L (0.191 gsm, 84 nm fiber) with total 0.57 gsm ➢ 8L (0.096 gsm, 349 nm fiber) with total 0.77 gsm FE for two filters with higher fiber basis weight ➢ 3.1 gsm (90%), pressure drop (18 Pa) ➢ 4.6 gsm (94%) pressure drop (26 Pa)	[80]

**Table 3:** Summary of face mask and respirator filtration experiments and testing practices using KCl particles.

Purpose of Study	Mask Material	Modeled Aerosol Particles	Experimental Aerosol Particle Generator	Notes on Filtration Efficiency	Reference
Investigated bilayer, beaded ENMs to prepare efficient lightweight respiratory filter media with less pressure drop. Focused on producing a continuous bead on string nanostructure at small basis weight levels (0.5 to 2 g/m^2^)	Single layer and Bilayer Electrospun nanofiber membrane (ENM)	KCl particles *dp* (0.3 to 5 μm)	Atomizer generated aerosols of KCl which flow through the rig at a controlled flow rate	Bilayer ENM with bead-free fibers on top and beads at bottom greatly improved FE (η = 95.7%) and reduced the pressure drop (Δ*P* = 137 Pa) The bilayer ENM performance (η = 95%; Δ*P* = 112 Pa) at small basis weight (0.5 g/m^2^) was on par compared to a commercial disposable respirator mask	[119]
To reduce the expulsion of small cough-generated aerosol particles into the environment, the study conducted an efficacy quantitative comparison of: N95 respirator. A medical procedure masks. A commercial 3-ply cloth face mask. A single- and double-layer fabric neck gaiter. A commercial disposable face shield as source control devices.	N95 medical respirator (3M model 1860). Medical grade (ASTM Level 3) procedure mask with ear loops (Kimberly-Clark model 47107). Cloth face mask with 3 layers of cotton fabric and ear loops (Hanes Defender). Fabric neck gaiter (FKGIONG Sun UV Protection Neck Gaiter, 95% polyester, 5% Spandex). Disposable face shield (Fisher Scientific # 19-181-600A).	Cough aerosol particles *dp* (0 to 7 μm)	Modified Greene and Vesley testing method was used. Cough aerosol simulator with a pliable skin head form to propel small aerosol particles. Aerosol particles were generated by nebulizing a solution of 14% KCl and 0.4% sodium fluorescein using a single-jet Collison nebulizer (BGI, Butler, NJ, USA) at 103 kPa (15 lbs./in2), passing the aerosol through a diffusion drier (Model 3062, TSI, Shoreview, MN, USA), and mixing it with 10 L/min of dry filtered air.	Cloth face mask collection efficiency 28% for the <0.6 μm particles. 76% for the 4.7 to 7 μm particles.Double-layer gaiter blocked 24% of the <0.6 μm particles. 76% of the 4.7 to 7 μm particles.	[120]

**Table 4:** Summary of face mask and respirator filtration experiments and testing practices using latex particles.

Purpose of Study	Mask Material	Modeled Aerosol Particles	Experimental Aerosol Particle Generator	Notes on Filtration Efficiency	Reference
Establish a method to evaluate the FE of mask materials under extreme conditions. Present a simple way to test the filter performance of mask materials using micro-droplets sized particles and microspheres with a challenged velocity of 44.4 m/s created by centrifugation (7500 rpm)	Surgical masks. Washed surgical masks. Cotton gauze masks (containing 4 layers of cotton gauze, 2 layers of nonwoven fabric filter and 2 layers of polypropylene filter, washed). 4 types of cotton from T-shirts. 3 types of silk. 3 types of linen, tissue paper and cotton gauze. Surgical masks and cotton gauze masks were purchased from a Japanese drug store	Micro-particle FE test: Blocking micro-droplet sized (starch particles). *dp* (0.7 to 70 μm), average 8.2 μm. Microsphere FE test and Microsphere-capturing test: Latex microspheres.*dp* (0.75 μm)	Micro-particle FE test and Microsphere FE test: Centrifugation (7500 rpm, equaling 44.4 m/sec.) for 20 sec. to mimic the velocity of a sneeze. Microsphere-capturing test: Microsphere solution (50 to 100 μL) added on top of the sample	Four layers of silk blockage efficiency: 93.8% of microspheres. 88.9% of starch particles. Gauze mask blockage efficiency: 78.5% of microspheres. 90.4% of starch particles. Two layers of cotton blockage efficiency: 74.6% of microspheres. 87.5% to 89.0% of particles. Other materials blocked: 53.2% to 66.5% of microspheres. 76.4% to 87.9% of particles except the 8 layers of gauze (36.7%).	[121]
Examined the efficiency of commonly worn masks in the developing world: Three types of cloth masks. One type of surgical mask.	Two commercially available N95 masks from two different manufacturers in the United States (Rigid Moldex model (2701) and a 3M model (8200). The Moldex mask (N95 mask2) and one of the cloth masks (cloth mask 1) had a plastic and latex exhalation valve	Lab-generated polystyrene latex (PSL) microsphere. Five monodispersed aerosol sphere size (30, 100, and 500 nm and 1 and 2.5 μm).	PSL were generated by a constant output atomizer (model 3076; TSI, Shoreview, MN, USA). PSL were in a colloidal solution of single-size latex spheres.	FE for cloth mask with an exhaust valve (80% to 90%) for the measured polystyrene latex (PSL) particle sizes. Two styles of commercially available fabric masks were the least effective with a FE (39% to 65%) for PSL particles. Performance increased with increased particle size. FE for cloth masks tested against lab-generated whole diesel particles (30, 100, and 500 nm) ranged from 15% to 57%	[122]

**Table 5:** Summary of face mask and respirator filtration experiments and testing practices using particles generated by incomplete or complete combustion.

Purpose of Study	Mask Material	Modeled Aerosol Particles	Experimental Aerosol Particle Generator	Notes on Filtration Efficiency	Reference
Developed a custom experimental set-up to measure the effectiveness of nine different respirators under real environmental conditions: Particle mass concentration below 2.5 μm (PM2.5). Particle number concentration (PNC). Lung Deposited Surface Area (LDSA). Black Carbon concentration (BC).	Nine low-cost respirators and commonly used by cyclists and pedestrians. N99 filter layer, carbon filter 2. Combination filter for chemical and particle filtration. Electrostatic filter. Filter FFP3 3. Filter FFP1 3. Respirator with no available data. Active carbon filter. Electrostatic and active carbon filter. Non-woven fabric filter	Ambient ultrafine aerosol particles including black carbon. PM2.5	Face mask performances were assessed in a typically traffic affected urban background environment in the city of Barcelona under three different breathing patterns in order to investigate the influence of flowrate in face mask effectiveness	Median face mask effectiveness: 48% in a range of 14% to 96% for PM2.5. 19% in a range of 6% to 61% for BC concentration. 19% in a range of 4% to 63% for PNC. 22% in a range of 5% to 65% for LDSA.	[123]
Test the FE of a range of masks sold to consumers in Beijing. Assessed mask effectiveness in reducing exposure to diesel exhaust particulates when worn by volunteers	Nine masks were purchased in China 3M8210 3M9001 3M9322 3M9501 3M9502 Green Shield Yi Jie PM2.5 Gucheng Yimeijian	Fine diesel exhaust particulates (PM2.5). Black carbon (BC) concentration (50 μg/m^3^)	Tests were conducted in a chamber in Edinburgh, UK. Tests were conducted in an exposure chamber supplied with air from a mixing chamber connected to a small diesel engine	Mean penetration (%) for each mask material ranged from 0.26% to 29%, depending on the flow rate and mask material. Average total inward leakage (TIL) of BC: 3% to 68% in the sedentary tests. 7% to 66% in the active tests. FE of a face mask does not necessarily translate into consistent exposure reduction for individuals	[124]
Aimed to build the first evidence base on the effectiveness of common materials used to protect communities from ash inhalation in volcanic crises. The respiratory protection (RP) materials were characterized and subjected to FE tests, which were performed with three challenges chosen as a low-toxicity surrogate dust of similar particle size distribution.	17 forms of RP, covering various types of cloth through to disposable masks: Used in occupational settings Communities are known to wear during volcanic crises	Three type of dusts: Ashes from Sakurajima (Japan) and Soufrière Hills. (Montserrat) volcanoes. Sluminum oxide (Aloxite). Two PM2.5 concentrations (1.5 and 2.5 mg/m^3^)	The particle-air suspension was generated using a Venturi nozzle, and a rotating table was loaded with the dust	Median FEs against volcanic for: N95-equiv. (>98%). N99-equiv. (>98%). PM2.5 surgical-Japan (>98%). Basic flat-fold-Indonesia (>98%). Two standard surgical masks (89% to 91%). All other materials (23% to 76%). No cloth materials (>44%). Folding a bandana resulted in better FE (40%; 3x folded) than single-layered material (29%). Wetting the bandana and surgical mask material did not improve FE overall	[125]
Presented a washable multilayer triboelectric air filter (TAF) for efficiently removing the PMs	Washable high-efficiency uncharged and charged triboelectric air filter (TAF). The TAF consists of five layers of the polytetrafluoroethylene (PTFE) and nylon fabrics	Smoke (<0.3 μm to >10 μm). Most of the particulate matter were <1 μm	The removal efficiency of the uncharged and charged TAF was performed in a 30 m3 lab. PMs were generated by burning cigarettes	After charging, the TAF has a removal efficiency of: 84.7% for PM0.5 (3.22 × times unchangred TAF). 96.0% for PM2.5 (1.39 × times unchangred TAF). The TAF is promising for fabricating a reusable and high-efficiency face mask	[76]
Developed a novel self-powered electrostatic adsorption face mask (SEA-FM) based on the poly (vinylidene fluoride) electrospun nanofiber film (PVDFESNF) and a triboelectric nanogenerator (TENG) driven by respiration (R-TENG)	A self-powered electrostatic adsorption face mask (SEA-FM) with a low pressure drop based on the RTENG and electrospun. Three PVDF-ESNFs with different electrospun times (30, 60, and 90 min)	Ultrafine particulates. *dp* (10 to 1000 nm)	The particulate matters used were generated by burning cigarettes because of the merits such as wide particulates size distribution from 10 nm to 10 μm, low price, and close to the existence of real environment particulates	On the basis of the RTENG, the SEA-FM showed that the removal efficiency of coarse and fine particulates was higher than 99.2 wt%. The removal efficiency of ultrafine particulates was 86.9 wt% after continually wearing for 240 min and a 30-day interval	[91]

**Table 6:** Summary of face mask and respirator filtration experiments and testing practices using liquid particles.

Purpose of Study	Mask Material	Modeled Aerosol Particles	Experimental Aerosol Particle Generator	Notes on Filtration Efficiency	Reference
Investigate: Aerosol FE of common household materials. Filters effects on flow characteristics in the surrounding flow regions. Compare results to a commercial surgical mask and an R95 mask	Cotton. Non-woven fabric (fabric 1). Microfiber cloth. HVAC filter. Shower curtain. Vacuum bag. Coffee filter. Material made up as: Single-layer. Two-layers. Three-layers	Liquid aerosol droplets. *dp* (1 to 4.7 µm). Particle Density (1 kg/m^3^)	Generated liquid aerosol droplets by six jet atomizer (TSI Model 9306) was used to aerosolize the Di-Ethyl-Hexyl-Sebacat (DEHS) fluid	FE for Shower curtain (74.4%) and HVAC filter (74.7%) had lower efficiency for all aerosol droplets sizes. Averaged FE of the combined multilayer materials increased from 4% to 15% when compared to single-layer materials. FE multilayer materials (>95%) at aerosol *dp* (2.42 µm). FE (>90%) for three-layer materials (cotton-coffee filter-cotton and cotton-coffee filter-fabric 1)	[6]
Demonstrated a simple optical measurement method to evaluate the efficacy of masks to reduce the transmission of respiratory droplets during regular speech. Compared the efficacy of different masks by estimating the total transmitted droplet count	14 commonly available masks or masks alternatives. One patch of mask material. A professionally fit tested N95 mask.	Water particles from a spray bottle	The front of the box had an 18 cm diameter hole for the speaker, large enough for a person wearing a mask to speak into the box but small enough to prevent the face (or mask) from reaching the light sheet	Some mask types approach the performance of standard surgical masks, while some mask alternatives, such as neck fleece or bandanas, offer very little protection	[11]
Used qualitative visualizations to examine the performance of face shields and exhalation valves in impeding the spread of aerosol-sized droplets	A face shield (similar in design to those used by healthcare workers in conjunction with masks and other protective equipment). An N95 mask with an exhalation valve located at the front	Droplets of distilled water and glycerin. *dp* (<10µm)	The setup consists of a hollow manikin head, where a cough/sneeze was emulated via a pressure impulse applied using a manual pump	The visualizations indicated: Face shields blocked the initial forward motion of the jet. Expelled droplets can move around the visor with relative ease and spread out over a large area depending on light ambient disturbances. Visualizations for a mask equipped with an exhalation port indicated that a large number of droplets pass through the exhale valve unfiltered, which significantly reduces its effectiveness as a means of source control	[126]
Showed from a fluid physics point of view and under different circumstances the type of masks can protect against droplet infection. Analyzed the flow blockage caused by surgical masks when coughing and qualified the effectiveness of different filter materials and masks to determine the protection ability against droplets. Attempted to prove the effect of gap flows at the edge of surgical and particle filtrating respiratory masks	Surgical face mask Hygienic mask Toilet paper Paper towel Coffee filter Microfibre cloth Fleece Vacuum cleaner bag FFP3 mask with valve Halyard H600	DEHS (Di-Ethyl-Hexyl-Sebacat) tracer particles. *dp* (0.1 to 2 μm)	The droplets were generated from DEHS with an aerosol seeding generator (AGF 2.0, Palas GmbH, Karlsruhe, Germany)	Mechanisms that include preventing a smear infection, applying adequate flow resistance to spreading virus in a room, and preventing inhalation of droplet, can be only achieved with FFP2/N95/KN95 or better particle filtering respirator mask	[127]

**Table 7:** Summary of face mask and respirator filtration experiments and testing practices using modeled bioaerosol particles.

Purpose of Study	Mask Material	Modeled Aerosol Particles	Experimental Aerosol Particle Generator	Notes on Filtration Efficiency	Reference
Evaluated the relative contributions of a mask, valve, and Micro Ventilator on aerosol FE of a new N95 respiratory face mask	N95-rated (16) respiratory face mask with Micro Ventilator	*Influenza A (H1N1) virus, strain A/PR/8/34* (from Charles River Laboratories (Horsham, MA, USA). *Rhinovirus type 14, strain 1059 (ATCC VR-284)* (from the American Type Culture Collection (Manassas, FL, USA)	Six-jet Collison nebulizer (Mesa Labs, Butler, USA) was filled with a measured amount of virus suspended in 0.1× Minimum Essential Medium (MEM). Virus was aerosolized and delivered into the upstream chamber using high-pressure air	FE (>99.7%) for each test mask configuration for exclusion of *influenza A virus*, *rhinovirus 14*, and *S. aureus.* FE (>99.3%) for paraffin oil and sodium chloride (surrogates for PM2.5)	[128]
Evaluated the FEs and microbial recovery rates of commercial filtering facepiece respirators against bioaerosols	Eight filtering facepiece respirators and one surgical mask were selected	Bioaerosols: *Staphylococcus epidermidis.* *Escherichia coli*	Bioaersols were released from a from a 6-jet nebulizer (Collison Nebulizer; BGI, Butler, NJ, USA) at 1.0 psig (Bioaerosol Spraying)	FE of each filtering facepiece respirator ranged from 82% to 99%, depending on the filtration grade	[129]
To conclude whether there is an effective mask for the population to wear in public that could easily be made during a medical face mask shortage using readily available materials. Test if the filter material of ePM_1_ 85% (ISO 16890) or F9 (EN 779:2012), similar to the American MERV 16 filter standards, could approach the filter capacity of an FFP2 mask	Two filter material types: ePM_1_ 85% (ISO 16890). F9 (EN 779:2012.	dp (0.3, 0.5, 1.0 and 5.0 µm). *Staphylococcus aureu* was used for testing EN 14683:2014 (surgical masks)	For the fit test: The face mask was equipped with an inlet to a tube. The flow was created through the tube, and the number of particles in the mask is counted	Fourteen of the 25 (combinations of) materials filtered at least 35% of 0.3-mm particles. Four of the materials proved hydrophobic, all commercially manufactured filters. Two models sealed the face. Twenty-two of the 25 materials were breathable at <0.7 mbar. None of the hydrophobic materials stayed intact after washing	[130]
Assessed household textiles to quantify their potential as effective environmental droplet barriers (EDBs). Using a bacterial-suspension spray simulation model of droplet ejection (mimicking a sneeze), the extent was quantified by which widely available clothing fabrics reduce the dispersion of droplets onto surfaces within 1.8 m (COVID-19 recommended minimum social distancing)	Six household textiles: 100% combed cotton (widely available, T-shirt material). 100% polyester microfiber 300-thread count fabric (pillow case). 100% cotton fabrics, two loosely homespun woven: 140GSM, 60 × 60-thread count. 115GSM, 52 × 48-thread count). 100% polyester common in sport jerseys (dry technology)	To simulate a cloud of droplets produced by a sneeze, a household spray bottles filled with an aqueous suspension of 12 probiotic cultured dairy product: *Lactobacillus lactis* *L. rhamnosus* *L. plantarum* *L. casei* *L. acidophilus* *Leuconostoc cremoris* *Bifidobacterium longum* *B. breve* *B. lactis* *Streptococcus diacetylactis* *Saccharomyces florentinus* 75 mL; 3 × 10^6−7^ cfu/mL, 25 mL Saliva 10^6−7^) in 1000 mL PBS (Fisher BP-399-1)	Spray bottle nozzles were adjusted to produce cloud and jet-propelled droplets that match a specific visual architecture of droplet formation. A high-volume trigger single-v-orifice nozzle sprayer was used (1.0 mL per stroke) with a 28/400 neck and 9-1/4-inch dip tube fitted with a filter screen (model PA-HDTS-EA, Mfr. Model # 922HL, Delta Industries, Inc.). The spray bottle ejected fluid with pressures that can reach 10 psi to create a short burst of fluid/jet and fan clouds of microdroplets	Spray experiments with “two-layers” (of 100%-combed cotton, common in t-shirts; and 100% polyester, in sports jerseys) Completely prevented the ejection of large macro-droplets (100% EnvDC prevention). Drastically reduced the ejection of micro-droplets by a factor of 5.16Log2, which is equivalent to a 97.2% droplet reduction (*p* < 0.020 vs. single-layers). The least-effective textile as single-layer (most breathable, 100%-cotton homespun-115 material) achieved a (90% to 99.998%) droplet retention improvement when used as two-layers (95% CI = 3.74–15.39 Log2). Two-layers of household textiles were as effective as medical masks preventing EnvDC, and that more breathable materials in ≥2-layers could be effectively used if individuals deem two-layer, “denser” textiles too air-restrictive	[131]
Measured the FEs of “N95 FFRs” including six N95 FFR models, three surgical N95 FFR models, and three SM models using testing methods: NIOSH NaCl aerosol. FDA particulate filtration efficiency (PFE). FDA bacterial filtration efficiency (BFE). Viral filtration efficiency (VFE) adapted by Nelson Laboratories from the ASTM F2101 method	N-series FFRs. Six NIOSH-approved. N95 FFR models. Three surgical N95 FFR models. Three SM models. Purchased from the United States Strategic National Stockpile or from respirator manufacturers known to have significant market share. The manufacturers and models in parentheses are: N95 FFRs-3M (Model 8210), 3M (Model 9210). Moldex(Model 2200), Kimberly-Clark (Model 62126). Sperian-Willson (Model SAF-T-FIT), and US Safety (N95B240). Surgical N95 respirators-3M (Model 1860). 3M (Model 1870) and Kimberly-Clark (Model 46727). SMs-3M (Model 1820), Kimberly-Clark (Model 47107) and Precept (15320)	NIOSH NaCl aerosol (Charge neutralized polydisperse sodium chloride, dp 0.022 to 0.259 μm). PFE (unneutralized 0.1 μm polystyrene latex (PSL) particles). BFE (not charge neutralized ∼ 3.0 μm size water droplet particles containing *Staphylococcus aureus* bacteria). VFE (unneutralized 3.0 μm size water droplet particles containing *bacteriophage phiX 174* as the challenge virus and *Escherichia coli* as the host)	NIOSH NaCl aerosol 2% (wt/vol) NaCl solution was aerosolized, charge neutralized and then passed through the convex side of a test sample properly sealed and placed into a filter holder), total load 200 mg of NaCl. PSL particles were suspended in water and the aerosol was generated using a particle generator (Model PG-100) (Particle Measuring Systems (PMS), Boulder, CO). BFE (suspension of *S. aureus* was aerosolized using a nebulizer to give a challenge level of 1700–2700 colony-forming units (CFU) per test as specified by the ASTM F2101 standard). VFE (suspension of phiX174 was aerosolized in a nebulizer and each test was performed with a challenge level of 1700-2700 plaque-forming units (PFU) with a MPS of 3.0 ± 0.3 μm for 2 min	N95 FFRs FE values: NIOSH NaCl method (98.15% to 99.68%) PFE (99.74% to 99.99%) BFE (99.62% to 99.9%) VFE (99.8% to 99.9%) Efficiencies by the NIOSH NaCl method were significantly (*p* ≤ 0.05) lower than the other methods. SMs showed lower efficiencies (54.72% to 88.40%) than “N95 FFRs” measured by the NIOSH NaCl method, while PFE, BFE, and VFE methods produced no significant difference	[89]
Developed a method for generating PPE that can be easily replicated at other sites for use when supplies are critically low, and use of locally manufactured masks with known bacterial filtration efficiency (BFE) ratings is logically superior to alternatives (like cloth masks or scarves)	Four different surgical wraps, all from the Medline GEM Series with a single and a double layer ply. Eight mask prototypes were constructed in a consistent tri-fold design from each type of GEM wrap and single or double material layers	All eight prototypes were sent to an environmental lab for: BFE testing. Latex particle FE testing. Delta P testing.		BFE rates depending on specific material and ply (83.0% to 98.1%): Two ply masks produced with Medline GEM 1, 2, and 3 materials (96.3% to 98.1%). One single ply mask separated prior to mask manufacture (83.0% to 97.7%). Particular FE rates (92.3% to 97.7%)	[132]

**Table 8:** Summary of face mask and respirator filtration experiments and testing practices using particles generated by human activities.

Purpose of Study	Mask Material	Modeled Aerosol Particles	Experimental Aerosol Particle Generator	Notes on Filtration Efficiency	Reference
Developed an airborne transmission simulator of infectious *SARS-CoV-2* containing droplets/aerosols produced by human respiration and coughs Assessed: The transmissibility of the infectious droplets/aerosols. The ability of various types of face masks to block the transmission.	Cotton masks Surgical masks N95 masks N95 (fit masks)	Droplets/aerosols produced by human. Infectious droplets/aerosol. Virus suspension (5 105 PFU [A to E], 1 108 PFU [F and G], 1 105 PFU [H], and 1 104 PFU [I])*dp* (5.5 ± 0.2 µm) Particle size percentages: 20% (<3 µm) 40% (3 to 5 µm) 40% (5 to 8 µm)	Airborne transmission simulator. Charged nebulizer with 6 ml of virus suspension at the viral doses in culture medium (without fetal calf serum) or diluted in phosphate-buffered saline to generate droplets/aerosols	Airborne simulation experiments showed that cotton masks, surgical masks, and N95 masks provide some protection from the transmission of infective *SARS-CoV-2* droplets/aerosols. Medical masks (surgical masks and N95 masks) could not completely block the transmission of virus droplets/aerosols even when sealed	[133]
Measured outward emissions of micron-scale aerosol particles by healthy humans performing various expiratory activities while wearing different types of medical-grade or homemade masks	Surgical mask (ValuMax 5130E SB). Unvented KN95 respirator (GB2626-2006). Homemade single-layer paper towel mask (Kirkland, 2-PLY sheet). Homemade single-layer t-shirt mask (Calvin Klein). Homemade double-layer t-shirt mask. Vented N95 respirator (NIOSH N95, Safety Plus, TC-84A-7448)	Micron-scale aerosol particles by healthy human. *dp* (0.3 to 20 μm)	Healthy Human Expiratory activities: Breathing (2 min). Talking (100–150 s). Coughing (30 s). Jaw Movement (1 min).	Outward particle emission rate for speaking and coughing: Surgical masks (Reduced by 90%). Unvented KN95 (Reduced by 74%). Outward particle emission rate for all expiratory activities: Homemade cotton masks (remained unchanged). Homemade single-layer t-shirt mask (Increased by 492%).	[134]
Explored the importance of respiratory droplet and aerosol routes of transmission with a particular focus on coronaviruses, *influenza* viruses, and rhinoviruses, by quantifying the amount of respiratory virus in exhaled breath of participants with medically attended ARIs and determining the potential efficacy of surgical face masks to prevent respiratory virus transmission	Surgical face mask (cat. no. 62356, Kimberly-Clark)	Respiratory droplets > 5 μm. Aerosol droplets ≤ 5 μm.	Breathing as normal during the collection, but (natural) coughing was allowed and the number of coughs was recorded by study staff. Participants were then invited to provide a second exhaled breath sample of the alternate type (for example, if the participant was first assigned to wearing a mask, they would then provide a second sample without a mask), but most participants did not agree to stay for a second measurement because of time constraints.	Detected coronavirus in samples collected without face masks: Respiratory droplets in 3 of 10 (30%) Aerosols in 4 of 10 (40%) No detection of any virus in respiratory droplets or aerosols collected from participants wearing face masks. In samples collected without face masks, *influenza* virus was detected: Respiratory droplet in 6 of 23 (26%). Aerosol in 8 of 23 (35%). There was a significant reduction by wearing face masks to 1 of 27 (4%) in detection of *influenza* virus in respiratory droplets, but no significant reduction in detection in aerosols. Results indicated that surgical face masks could prevent transmission of human coronaviruses and *influenza* viruses from symptomatic individuals. Surgical face masks significantly reduced detection of *influenza* virus RNA in respiratory droplets and coronavirus RNA in aerosols, with a trend toward reduced detection of coronavirus RNA in respiratory droplets	[3]

**Table 9:** Summary of face mask and respirator filtration experiments and testing practices using silicon dioxide, radioactive, and fluorescent aerosol particles.

Purpose of Study	Mask Material	Modeled Aerosol Particles	Experimental Aerosol Particle Generator	Notes on Filtration Efficiency	Reference
Ascertained the performance of 11 common household fabrics at blocking large, high-velocity droplets, using a commercial medical mask as a benchmark. Assessed the breathability (air permeability), texture, fiber composition, and water absorption properties of the fabrics. Developed a method of quantifying the effectiveness of fabrics at blocking large droplets containing 100 nm diameter nanoparticles which serve as a mimic for viruses in terms of size	One medial mask as control 11 common household fabrics: Medical mask (FM-EL-style, polypropylene, non-woven). Fabric (used shirts, under shirts, T-shirt, new bed sheet, new quilt cloth, variable % of cotton and polyester, woven or knit). Fabric (new dish cloth, 80% polyester, 20% polyamide, napped). Fabric (used shirt, silk, woven)	Fluorescent nanoparticles (beads). *dp* (25 nm, 300 nm)	Used a metered-dose inhaler (HFA-propelled, 210 sprays, GlaxoSmithKline) and loaded its nozzle with 10 μL of distilled water to generate droplets. Droplets were generated using a suspension of 100 nm-diameter red fluorescent beads (ex/em 580/605 nm, Invitrogen, catalog #F8801) diluted in distilled water	Blocking FE Values: Most fabrics (median values > 70%). Two layers of highly permeable fabric (>94%) similar to that of medical masks, while being approximately twice as breathable	[8]
Designed an in vitro model using various facepieces to assess their contribution to exposure reduction when worn at the infectious source (Source) relative to facepieces worn for primary (Receiver) protection, and the factors that contribute to each	Fitted (SecureFit™) surgical mask and an N95-class filtering facepiece respirator (commonly known as an ‘N95 respirator’) with and without a Vaseline-seal	Nebulizer and exhaled radioactive aerosols	Aerosol released from the source by tidal breathing or cough. Two manikins were connected to a Harvard ventilation pump (Harvard Apparatus SN No. A52587; Millis, MA, USA)	With cough, source control (mask or respirator on Source) was statistically superior to mask or unsealed respirator protection on the Receiver (Receiver protection) in all environments. To equal source control during coughing, the N95 respirator must be Vaseline-sealed.During tidal breathing, source control was comparable or superior to mask or respirator protection on the receiver	[135]
Addressed concerns that publication of only the ideal FE of materials in perfectly sealed settings can give mask wearers a false sense of security when venturing into areas of high exposure risk. Evaluated the FE of respirators, masks, and filter media against the smallest possible virus-carrying particulates	Several commercially available masks and respirators were tested as received without further modification: Cotton 1 Layer Dust Mask #4 Coffee filter Cotton 2 layers Shop towel Filtrete 1500 Surgical wrap N95 1 layer Medical mask ShopVac KN95 N95 2 layers 3M 8511 FTR467 ULPA	Polydisperse silicon dioxide nanoaerosol. *dp* (60 nm and 125 nm)	Polydisperse silicon dioxide nanoaerosol was generated in an electropolished steel environmental chamber designed according to the specifications of ANSI/CAN/UL 2904, measuring 4′ × 3′ × 3′ with cleanroom air (background total particulate concentration <10 particles/cm^3^) injected at a rate sufficient to induce one full chamber air exchange per hour	Results demonstrate the importance of fit on FE. Wearing a homemade mask can and does significantly reduce virion-sized particulate exposure (as reported worn filtration efficiencies of 15% to 40%). Homemade masks cannot provide the level of protection measured and more commonly reported in ideal-fit scenarios.For 3M 8511 and KN95: FE (>98%). When fit to the headform, the FE dropped to less than 40%, slightly better than the fitted cotton mask. Insertion of the extra layer to cotton masks: Did not significantly improve the cotton mask performance for most tested materials. In most cases, the cotton mask offered practically equivalent levels of protection without the insertion of the extra layer	[13]

**Table 10:** Decontamination and regeneration using thermal disinfection (T ≡ Temperature, RH ≡ Relative Humidity, D ≡ Detect, and ND ≡ Not Detect).

Method	Modeled Aerosol	Material	Decontamination Condition(s)	Filtration Performance	Microbial Activity	Reference
Thermal Disinfection	*SARS-CoV-2*; *Human coronavirus NL63 (Hcov-NL63)* *Chikungunya virus*	N95	Heating block T (75–85 °C) Thermal Loading (20–30 min) RH (100%)	Steady FE at: 20 cycles of 75 °C for 30 min/cycle 85 °C for 20 min/cycle at 100% RH. 75 °C for 30 min reduced viral titers by 3.5 log10-fold at 60% RH. Sharp drop in FE (90%) at T (125 °C) at fifth cycle	*SARS-CoV-2* virus: ND at T (75 to 95 °C) and RH (100%) CHIKV-181/25: ND at T (85 or 95 °C) HCoV-NL63 titers below LOD at T (85 °C) for 20 minHCoV-NL63 titers: D at T (95 °C) for 5 min	[137]
	*Human Adenovirus Type 2* (AdV; Adenoviridae) *Rotavirus OSU* (RV; Reoviridae) *Tulane virus* (TV) *Porcine transmissible gastroenteritis virus* (TGEV)	N95 (1860, 3M)	Dry heat using electric cooker. T(100 °C) Thermal loading (50 min) RH (5%)	FE (97%) and fit testing did not degrade after 20 cycles and thermal loading (50 min) using dry heat treatment Pressure drop was not significant	Thermal energy conveyed to viruses by dry heat T (100 °C) and thermal loading (50 min) resulting in: >5.2-log10 reduction for TV >6.6-log10 reduction for RV >4.0-log10 reduction for AdV >4.7-log10 reduction for TGEV	[138]
	*S. aureus*	Surgical mask	Dry heat T (100 °C), thermal loading (15 min) Steam-used steamer cooker at T(100 °C), thermal loading (10 min) Water bath at T(100 °C), thermal loading (10 min) Autoclave T(121 °C), thermal loading (20 min)	All treatments showed a decrease in FE Dry heating did not show significant decrease after 3 cycles. Maintained FE (95%) Dry heat did not show significant effect on mask hydrophobicity Boiling, steaming, and autoclaving caused structural changes in mask material	Deactivation of bacterial activity (< 4-log reduction) for all heating methods	[139]
	*Escherichia coli* *Bacillus subtilis*	Surgical mask FFP1, FFP2, FFP3	Steam used was less than 100 °C at normal atmospheric pressure	FE decreased (98.86% to 97.58%) at particle size (50 nm) FE was affected by a slight decay in the surface potential Steam changed morphology of mask sample	Deactivated *E. coli* by 100% at thermal loading (90 min)	[136]
	*Vesicular stomatitis virus* *Indiana serotype* (VSV) *SARS-CoV-2*	N95 (3M VFlex 1804, Aura 1870, 1860, 8210, and 9210)	Autoclave at T(121 °C), thermal loading (15 min) Air removal, exposure, and drying, leading a total of 40 min per cycle	Functional integrity was kept for all masks after 10 cycles besides 3M 1860 and 8210 (molded) models which failed	>6-log 10 reduction of infectious virus	[140]
	*Porcine respiratory coronavirus* (PRCV)	Surgical masks KN95	Dry heat at T(102 °C), thermal loading (60 min)	N/A	Infectivity of virus reduced by 2 orders of magnitude	[141]
	*Escherichia coli* *SARS-CoV-2*	N95 (3M-1860S,8110S,8210S,9105S)	T (70 °C) RH (0% to 70%) Thermal loading (60 min)	Post treated respirators showed >95% FE after 10 cycles Heating did not affect the structural integrity of the mask after 10 cycles at RH (0% and 50%)	Infectious virus ND after dry heating at T(70 °C), thermal loading (60 min) Inactivated *E. coli* after dry heating at T(70 °C), thermal loading (60 min), RH (50%)	[142]
	*E.Coli* NaCL aerosol was used for FE test	N95 KF94	Drying oven T (90 °C) Thermal loading (60 min) for N95 and KF94 grade respirators	Treatment did not show any significant effect on FE (<2%)	82% deactivation of the bacteria	[145]

**Table 11:** Decontamination and regeneration using microwave irradiation (MWI).

Method	Modeled Aerosol	Material	Decontamination Condition(s)	Filtration Performance	Microbial Activity	Reference
Micro-waving	*Escherichia coli* *Bacillus subtilis*	Surgical mask FFP1, FFP2, FFP3	400 W Thermal loading (10 min)	MWI did not show any significant effect on surface potential (no significant effect on FE) Morphology was affected over a long period of microwaving	Deactivated *E. coli* and *B. subtilis* by 100% (>4-log 10 reduction) in 10 min	[136]
	Poly-disperse sodium chloride aerosol	Surgical mask N95	1100 W (750 W/ft^3^) Loading 2 min (1 min for each side of the mask) Mask were cooled at ambient temp between trials	No FE drop for 2 min exposure. N95 filter melted after 4 min forming visible holes	N/A	[144]
	Polydisperse sodium chloride aerosol	Surgical mask N95	1100 W (750 W/ft^3^) Loading: 2 min total exposure duration at a power setting of 10	Partial separation of mask foam cushion and melting of head straps in mask samples	N/A	[143]
	*E. coli* NaCl aerosol was used for FE test	N95 KF94	750 W Loading: 1 min on both sides after removal of the metal nose clips	Treatment did not show any significant effect on filtration efficiency (<2%)	82% deactivation of the bacteria	[145]

**Table 12:** Decontamination and regeneration using UV irradiation.

Method	Modeled Aerosol	Material	Decontamination Condition(s)	Filtration Performance	Microbial Activity	Reference
UV irradiation	*Escherichia coli* *Bacillus subtilis*	Surgical mask FFP1, FFP2, FFP3	254 nm wavelength Loading: 5 min (126 mj/cm^2^)	No significant effect on FE	100% deactivation of *E. coli* UV+MW showed 100% inactivation of *E. coli* and *B. subtilis* in 5 min UV + 4/8/12 min MWI	[136]
	*S. aureus*	Surgical mask	254 nm UV irradiation Loading: 5 min	FE (>95%) No significant FE drop after 3 treatment cycles.	Eliminates all bacterial activity on exposure to 450 microW/cm^2^ for 10 min due to irradiation penetration limitations	[139]
	Poly-dispersed sodium chloride	Surgical mask N95	40 W (UV light intensity 0.18 to 0.20 mW/cm^2^) Loading: 5 min exposure on each side.	No particle filtration efficiency drop	N/A	[144]
	Porcine respiratory coronavirus (PRCV)	Surgical masks KN95	5.5 W (UV lamp) Loading: 2–4 min	N/A	Infectivity of virus reduced by 3 orders of magnitude	[141]
	*E. coli* NaCl aerosol was used for FE test	N95 KF94	10 W (UV lamp) Loading: 60 min on both sides of the respirator	No significant effect on FE	82% deactivation of the bacteria	[145]
	NaCl particles were used for FE test	N95 Surgical mask	Variable UGVI dosage	950 j/cm^2^ showed a slight decrease in particle penetration FE (up to 1.25%)	N/A	[146]

**Table 13:** Decontamination and regeneration using chemicals.

Method	Modeled Aerosol	Material	Decontamination Condition(s)	Filtration Performance	Microbial Activity	Reference
Chemicals	Porcine respiratory coronavirus (PRCV)	Surgical masks FFRs	Vaporized hydrogen peroxide (59% H_2_O_2_ with peak VHP concentration of 750 ppm)	N/A	Infectivity of virus reduced by 1 order of magnitude	[141]
	*Escherichia coli* *Bacillus subtilis*	Surgical mask FFP1, FFP2, FFP3	75% ethanol Loading: 2 min	Showed significant effect in surface potential. Partial change in morphology	Inactivates bacteria completely	[136]
	*Vesicular stomatitis virus*, *Indiana serotype* (VSV) or *SARS-CoV-2*	N95 (3M VFlex 1804, Aura 1870,1860,8210 and 9210)	Ethylene oxide-(1hr exposure)Low temperature hydrogen peroxide gas plasma-47 min cycle Vaporous hydrogen peroxide (VHP) with peak VHP concentration of 750 ppm Peracetic acid fogging	EtO maintained FE after 3 cycles for all tests. LT-HPGT-treated masks failed after the first cycle.VHP and PAF treatments maintained both functional and structural integrity after 10 cycles. Could not validate the effectiveness against *SARS-CoV-2*	> 6 log 10 reduction of infectious virus for all methods.	[140]
	*E. coli* NaCl aerosol was used for FE test	N95 KN94	sodium hypochlorite (NaClO) (5.5%) Sodium hydroxide (NaOH) (0.3%) and water 5%(v/v) Ethanol solution immersion of 70% (v/v) for 10 min Isopropanol immersion 100% for 10 min	Significant decrease in FE (up to 28%) in IPA and EtOH	100% deactivation of the bacteria	[145]

**Table 14:** Decontamination and regeneration using laundry detergent.

Method	Modeled Aerosol	Material	Decontamination Condition(s)	Filtration Performance	Microbial Activity	Reference
Detergent-laundering	*S. aureus*	Surgical mask	Household detergent (Ultra Axion) 0.55 *w/v* was prepared with DI water. Sample soaked in water for 30 min	Showed significant decrease in FE after first cycle	Did not show any effect on bacterial activity	[139]
	NaCl aerosol	N95 KN94	Laundering was done with and without detergent: Without detergent: agitation speed of 90 rpm at 24C for 10 min, with 2 repeats at 3 min With detergent: 1 L of 0.1 wt% aq. detergent solution at 24C and 90 rpm for 10 min	Without detergent: No significant change in FE With detergent: Significant decrease in FE (>23%)	N/A	[145]

**Table 15:** Different types of face masks and common materials used.

	Surgical	N95	Others
Mask Type	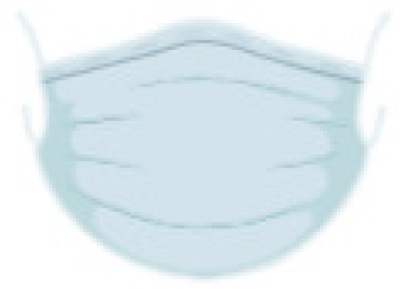	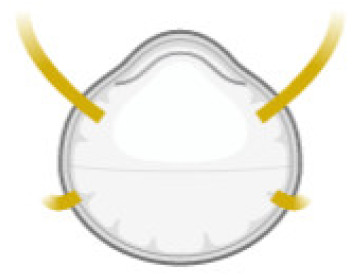	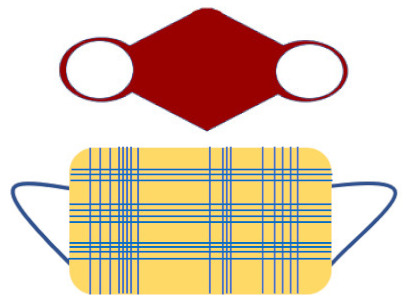
Common Materials	Polypropylene Polystyrene Polycarbonate Polyethylene Polyester	Polypropylene Cellulose PVDF PTFE	Cotton Chitosan Polyurethane Natural fiber

## Data Availability

The data presented in this study are based on a literature review of published materials and are available on request from the corresponding author.
